# Simultaneous quantitative profiling of clinically relevant immune markers in neonatal stool swabs to reveal inflammation

**DOI:** 10.1038/s41598-021-89384-0

**Published:** 2021-05-13

**Authors:** Veronika Vidova, Eliska Benesova, Jana Klanova, Vojtech Thon, Zdenek Spacil

**Affiliations:** Faculty of Science, RECETOX Centre, Masaryk University, Kamenice 753/5, Pavilion D29/418, 625 00 Brno, Czech Republic

**Keywords:** Diseases, Chemistry, Analytical chemistry, Biochemistry, Chemical biology, Biomarkers, Predictive markers, Prognostic markers, Peptides, Proteins, Proteomics

## Abstract

An aberrant immune response developed early in life may trigger inflammatory bowel disease (IBD) and food allergies (e.g., celiac disease). Fecal levels of immune markers categorize an inflammatory response (e.g., food allergy, autoimmune) paralleled with the initial microbial colonization. The immunoaffinity assays are routinely applied to quantify circulating immune protein markers in blood/serum. However, a reliable, multiplex assay to quantify fecal levels of immune proteins is unavailable. We developed mass spectrometry assays to simultaneously quantify fecal calprotectin, myeloperoxidase, eosinophil-derived neurotoxin, eosinophil cationic protein, alpha-1-antitrypsin 1, and adaptive immunity effectors in 134 neonatal stool swabs. We optimized extraction and proteolytic protocol and validated the multiplex assay in terms of linearity of response (> 100; typically 0.04 to 14.77 µg/mg of total protein), coefficient of determination (R^2^; > 0.99), the limit of detection (LOD; 0.003 to 0.04 µg/mg of total protein), the limit of quantification (LOQ; 0.009 to 0.122 µg/mg of total protein) and robustness. The median CV of intra- and interday precision was 9.8% and 14.1%, respectively. We quantified breast milk-derived IGHA2 to differentiate meconium from feces samples and to detect the first food intake. An early life profiling of immune markers reflects disrupted intestinal homeostasis, and it is perhaps suitable for pre-symptomatic interception of IBD and food allergies.

## Introduction

Inflammatory bowel disease (IBD) and food allergies are a highly prevalent and diverse group of intestinal disorders, potentially resulting in chronic inflammation of the gastrointestinal tract (e.g., Crohn's disease, ulcerative colitis, and celiac diseases). IBD is an umbrella term for disorders with unknown etiology affecting 6.8 million people globally^1^. In Western countries, as high as 10% of the population reacts abnormally to a food allergen, with the highest prevalence among younger children^2^. The global prevalence of celiac disease was 1.4%, significantly greater in children than adults^3^. However, specific causes of food allergies are challenging to explore, and the prevalence is likely underreported^4,5^.

The early life or even prenatal exposure of the naïve immune system to human gut microbiota plays a prominent role in immunomodulation, potentially resulting in an inappropriate immune response and disorders^6,7^. The mode of delivery (i.e., cesarean section or vaginal birth), nutritional and lifestyle factors (e.g., breastfeeding, hygiene, probiotics), or medication (e.g., antibiotics) shapes the composition of the human gut microbiota, influencing the response of the immature immune system and homeostasis^8–10^. Naturally-born neonates benefit from early colonization by the vaginal microbiome^11,12^. On the contrary, adverse influence is attributed to the early colonization by nosocomial or skin microbiota after cesarean section^13,14^. Breastfeeding reportedly prevents allergies, asthma, and infections^15^. Mucosal immunoglobulins, prebiotics, and other breast milk components protect against disease and allergies via modulation of developing immune system and intestinal barrier function. Mucosal epithelial cells are arranged into a tight junctional complex during intestinal maturation and later form the permeable barrier between the intestinal lumen and the *lamina propria*^16^*.* In autoimmune diseases, the presence of allergens and pathogens causes intestinal inflammation and disruption of epithelial cells. Neutrophils, monocytes, eosinophils, white blood cells, and blood protein occur in the stool due to the intestinal barrier's lower integrity, causing inflammation.

The naïve immune system's inflammatory response traced via specific immunological markers in meconium or first feces can reveal a disease condition. Fecal calprotectin (CAL1, CAL2) and myeloperoxidase (MPO) indicate neutrophilic inflammation (i.e., pathogen-induced response, autoimmune reaction). Calprotectin levels in adult patients correspond with the progression from irritable bowel syndrome (IBS) to chronic IBD^17^. Fecal MPO in adults is reportedly a parameter of IBD^18^ and ulcerative colitis severity^19^. The correlation between calprotectin and MPO in neonates was demonstrated^20^. Fecal eosinophil-derived neurotoxin (EDN) and eosinophil cationic protein (ECP) are often seen in a patient with food allergic colitis and typically present in children with an atopic family history before the age of two years^21^. High alpha 1-antitrypsin 1 (A1AT-1) and immunoglobulin A1 (IGHA1) levels point to the disrupted intestinal barrier function in toddlers^22^. The analysis of inflammatory markers in a neonatal cohort can perhaps later provide information on IBD and allergy development. Fecal immunoglobulin A2 (IGHA2) derived from breast milk distinguishes meconium from the first feces^23^ to assess the nutritional influence on the colonization by intestinal microbiota.

Serum ECP levels determined by immunoassays^24^ are diagnostic for eosinophil inflammatory activity in asthma and allergies to estimate disease severity. Fecal ECP, EDN, and MPO are typically quantified in radioimmunoassay or enzyme-linked immunosorbent assay (ELISA)^25^. Skarzynska et al. applied ELISA to quantify MPO in the meconium of healthy neonates (n = 80) and determined concentrations between 0.02 and 8.8 µg/g of meconium, an average of 1.8 µg/g^26^. Roca et al. determined the average concentration of fecal EDN and calprotectin at 7.4 µg/g and 910.3 µg/g in 174 healthy toddlers (0–12 months)^27^. Clinically relevant calprotectin levels are qualified via ELISA^28^ and A1AT levels via nephelometry^29^ in feces. Meconium A1AT levels determined in 19 healthy neonates were 3720 µg/g on average^30^. The quantification of fecal immunoglobulin A employs radial immunodiffusion or ELISA^31,32^. Meconium A1AT decreases, and immunoglobulin A content increases within the initial days of life^32^.

**Figure 1:**
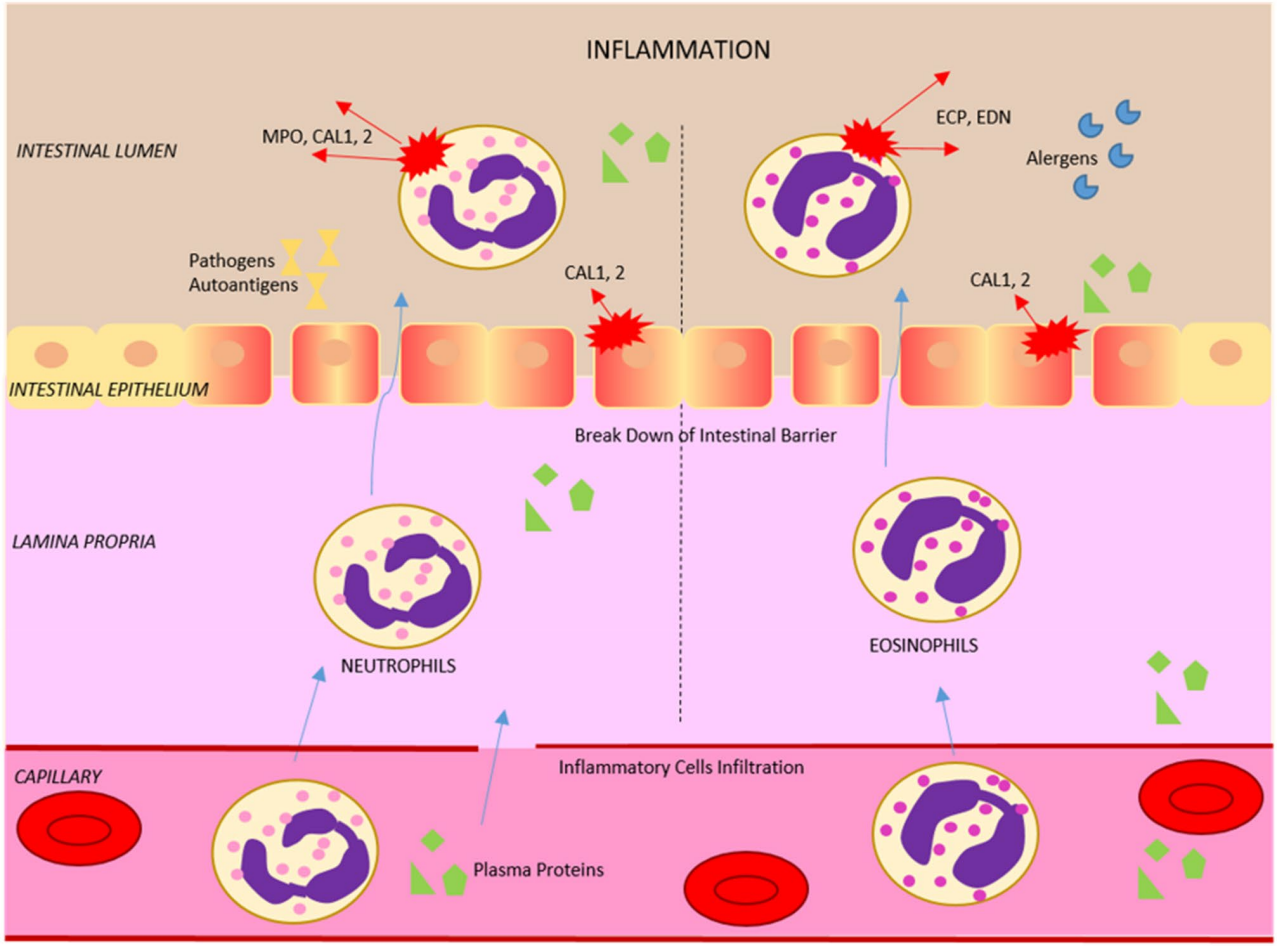
Scheme of immune protein markers' role in the intestinal inflammatory response, barrier function, and homeostasis.

Our study presents the multiplex quantification of inflammatory proteins in stool using ultra-high-performance liquid chromatography (UHPLC) and tandem mass spectrometry (MS/MS) in selected reaction monitoring (SRM) mode. The concept of UHPLC-SRM targeted proteomics is widely applicable in science, with clear advantages over immunoaffinity assays^33–36^. We are the first to present an SRM proteomics protocol for absolute quantification in neonatal meconium and feces swabs. The application towards a panel of clinically relevant markers (Table 1) reflecting the intestinal mucosal barrier homeostasis. The multiplex assay is suitable for classifying inflammatory response and potentially reveals a propensity of IBD and food allergies (Fig. 1)^22,32,37^.

**Table 1:** Immune protein markers were assayed in meconium and feces. The proteotypic surrogate peptides for absolute quantification. The position of stable isotope-labeled arginine (R*; ^13^C_6_H_14_O_2_^15^N_4_; + 10 Da mass shift) or lysine (K*; ^13^C_6_H_14_O_2_^15^N_2_; + 8 Da mass shift) in the proteotypic sequence of internal standard synthetic peptides marked in bold.

Protein number	Gene name	Name	Abbreviation	Proteotypic sequence
P01009-1	SERPINA1	Alpha-1-antitrypsin isoform 1	A1AT-1	AVLTIDE**K**
P01876	IGHA1	Immunoglobulin heavy constant alpha 1	IGHA1	TPLTATLS**K**
P01876 + P01877	IGHA1 + 2	Immunoglobulin heavy constant alpha 1 and 2	IGHA1 + 2	SAVQGPPE**R**
P01877	IGHA2	Immunoglobulin heavy constant alpha 2	IGHA2	DASGATFTWTPSSG**K**
P12724	RNASE3	Eosinophil cationic protein	ECP	NQNTFL**R**
P10153	RNASE2	Eosinophil-derived neurotoxin	EDN	DPPQYPVVPVHLD**R**
P05164	MPO	Myeloperoxidase	MPO	VVLEGGIDPIL**R**
P05109	S100-A8	Calprotectin 1	CAL1	ALNSIIDVYH**K**
P06702	S100-A9	Calprotectin 2	CAL2	DLQNFL**K**
				LGHPDTLNQGEF**K**

## Methods

### Chemicals

Proteotypic peptides selected as protein surrogates are in Table 1. Stable isotopically labeled (SIL) peptide standards with C-terminal arginine (R*; ^13^C_6_H_14_O_2_^15^N_4_; + 10 Da mass shift) or lysine (K*; ^13^C_6_H_14_O_2_^15^N_2_; + 8 Da mass shift) were custom synthesized (SpikeTides™ L and TQL, JPT Technologies, Berlin, Germany). We used SIL peptides (SpikeTides™ L) for assay development and optimization. SIL synthetic peptides extended at C-terminus with a trypsin cleavable tag (SIL-TCT, SpikeTides™ TQL) were used as internal standards for protein quantification. The trypsin cleavable tag consists of a tetrapeptide that incorporates a nitrotyrosine residue^38^. Sequences of all proteotypic peptides used for protein quantification are in Tables 1 and 2. Trypsin gold was from Promega (cat. #V5280). LC–MS grade acetonitrile (ACN, cat. #00136878) and isopropyl alcohol (IPA, cat. #16267802) was from Biosolv (Valkenswaard, The Netherlands). Formic acid (FA, cat. #94318), ammonium bicarbonate (ABB, ≥ 99.5%; cat. #09830), iodoacetamide (IAA, cat. #I6125), and sodium deoxycholate (SDC, ≥ 98%; cat. #30970) were from Sigma Aldrich (St. Louis, MO). Dithiothreitol (DTT, cat. #20290) and BCA protein assay kit (cat. #23225) were from Thermo Fisher Scientific (Waltham, MA). The deionized water was produced in Simplicity 185 ultrapure water system (Merck Millipore corp. Billerica, MA).

**Table 2:** The validation parameters of assessed proteotypic peptides, i.e., the linearity of response, assay range, LOD, and LOQ. All concentrations in (nM) for peptide surrogates and in (ug/mg) for target protein in total protein (by BCA)**.** The position of stable isotope-labeled arginine (R*; ^13^C_6_H_14_O_2_^15^N_4_; + 10 Da mass shift) or lysine (K*; ^13^C_6_H_14_O_2_^15^N_2_; + 8 Da mass shift) in the proteotypic sequence of internal standard synthetic peptides marked in bold.

Protein	Internal standard peptide sequence	Concentration added to sample (nM)	R^2^	LOD		LOQ		Range		Calibration levels
				(µg/mg)	(nM)	(µg/mg)	(nM)	(µg/mg)	(nM)	
A1AT-1	AVLTIDE**K**	100	0.992	0.003	0.20	0.009	0.62	0.01–28.27	1–2000	7
IGHA1	TPLTATLS**K**	100	0.997	0.010	0.86	0.030	2.62	0.05–22.77	4–2000	9
IGHA1 + 2	SAVQGPPE**R**	100	0.991	0.024	2.10	0.071	6.35	0.04–22.45	4–2000	9
IGHA2	DASGATFTWTPSSG**K**	120	0.998	0.037	3.31	0.111	10.04	0.11–22.13	10–2000	7
ECP	NQNTFL**R**	100	0.999	0.004	0.69	0.012	2.08	0.02–3.89	4–700	7
EDN	DPPQYPVVPVHLD**R**	100	0.993	0.014	2.58	0.043	7.81	0.02–3.33	4–600	8
MPO	VVLEGGIDPIL**R**	100	0.998	0.040	1.63	0.122	4.93	0.10–49.39	4–2000	7
CAL1	ALNSIIDVYH**K**	100	0.991	0.005	1.39	0.014	4.22	0.03–6.55	10–2000	8
CAL2	DLQNFL**K**	100	0.991	0.007	1.77	0.020	5.37	0.04–7.40	10–2000	8
	LGHPDTLNQGEF**K**	100	0.995	0.037	9.96	0.112	30.17	0.04–7.40	10–2000	6

### Meconium and feces swabs

Under the Declaration of Helsinki, the study was approved by the Committee for Ethics of CELSPAC: TNG (CELSPAC/EK/4/2016) at University Hospital Brno, the Czech Republic. The methods used in the study and described below were carried out following the relevant guidelines and regulations. The authors confirm that the data supporting this study's findings are available within the article and supplementary materials. Informed consent was obtained from a parent and/or legal guardian for all study participants and archived. The cohort study included only singleton pregnancies. Trained hospital personnel collected feces swabs from neonates (n = 134) during the first, second, or third day following the birth using FLOQSwabs (cat. #520CS01, Copan, Italy). Meconium or feces were scooped up from a conventional diaper using a swab, then the swab was placed inside a 2 ml vial, and the handle was broken off. The vial was sealed, immediately placed in a – 80 °C freezer, and stored until pre-analytical sample processing and analysis. The study subjects were female (n = 56) and male (n = 78) neonates at gestation age (weeks + days) ranging from 38 + 2 to 41 + 3. An average birth weight (g) 4394.1 ± 427.0 SD ranged from 2470 to 4900 g. Samples were from neonates delivered vaginally (n = 114) or via cesarean section (n = 20). Demographic and clinical characteristics of all study subjects and information on sample collection are listed in Table S1.

### Protein extraction protocol

We precipitated proteins by adding 1 ml of 80% IPA to the sample and orbital shaking (5 min, 1600 rpm). We centrifuged samples (2 min, 12,000 × g) and removed 50 µL of supernatant. The residual sample volume (950 µL), including the swab, was dried in speed vac overnight (minimum 6 h). Dried samples were reconstituted in 1500 µL of buffer (50 mM ABB with 5 g/l SDC) and homogenized (Benchmark Scientifics, Bead Blaster 24 homogenizer, 4 pulses × 30 s; 4 m/s; inter-time 10 s; ambient temperature). Next, we centrifuged samples (3000 × g; 10 min), transferred a supernatant (500 µL) into a clean vial, and centrifuged the vial again (12,000 × g; 5 min). After the second centrifugation, the supernatant (400 µL) was transferred into another clean vial for the trypsin digestion of extracted proteins. Total protein concentration in 134 neonatal meconium or feces swabs extracts (Table S2) were assessed in 10 µL of protein extract using the BCA assay (cat. #23225). We subjected the protein extract (50 µL) to the trypsin digestion protocol.

### Sample fresh weight total protein content

We prepared quality control (QC) material pooling fresh meconium and feces samples from 12 randomly selected neonates. Various QC material amounts were accurately weighed (25, 50, 75, 100, 125, 150 mg) on a swab in triplicate. We extracted proteins from feces swabs as described above and determined the protein content in protein extracts using the BCA assay (Table S3). We established the correlation between the stool sample fresh weight and total protein content and used QC swabs for method validation and reproducibility assessment.

### Trypsin proteolytic protocol

We reduced and alkylated protein extracts (50 µL) by adding 5 µL of 200 mM DTT (10 min at 95˚C) and consequently adding 5 µL of 400 mM IAA (30 min at ambient temperature in the dark). We added a working solution (10 µL; 500–600 nM) of the SIL-TCT peptide internal standards into a sample. Next, we added trypsin (3 µL; 1 µg/µL) approximately in the ratio of 1:70 to the total protein content and incubated samples at 37 ˚C (orbital shaking 200 rpm). We quenched the trypsin digestion after 5 h by adding 200 µL of 2% FA and peptides were purified using solid-phase extraction (SPE, Oasis HLB prime; 96-well plate format, 30 mg; Waters, Milford, MA). Samples were loaded on SPE, washed with 300 µL of 2% FA, and eluted with 200 µL 50% ACN with 2% FA. SPE eluates were dried in speed vac, reconstituted in 50 µL 5% ACN with 0.1% FA, resulting in 100–120 nM SIL-TCT internal standard concentrations in the sample (Table 2), and analyzed by UHPLC/SRM-MS.

### Proteolysis time optimization

Protein extracts from individually extracted QC swabs (n = 3) were combined (1200 µL) and divided into 21 identical aliquots (50 µL) to prepare the time-lapse experiment in triplicate. We did not add trypsin to samples without incubation (time 0 h), spiked with proteotypic SIL peptides (added 10 µL of 1.5 µM mixed working solution, all proteotypic sequences listed in Table 1). Trypsin was added to all incubated samples, spiked with SIL-TCT internal standards (all proteotypic sequences listed in Table 1). We quenched the trypsin digestion after 1, 3, 5, 17, 20, and 24 h of incubation. The 2% FA (200 µL) was added to all samples, followed by SPE processing and UHPLC/SRM-MS analysis. The reproducibility of trypsin digestion was tested using SIL-TCT peptides SAVQGPPER, DASGATFTWTPSSGK, TPLTATLSK, AVLTIDEK, DLQNFLK, LGHPDTLNQGEFK, ALNSIIDVYHK, DPPQYPVVPVHLDR, NQNTFLR, and VVLEGGIDPILR.

### Selected reaction monitoring mass spectrometry protein assays

Samples were injected (2 µL) on the UHPLC system (1260 series Agilent, CA) equipped with an analytical column (C_18_ Peptide CSH; 1.7 µm, 2.1 mm i.d. × 100 mm; cat. #186006937; Waters, Milford, MA) thermostated at 40 °C. The mobile phase consisted of solution A (0.1% FA in water) and solution B (0.1% FA in ACN). The flow rate was 300 µL/min, and the gradient elution program consisted of analytical (0–30.9 min) and re-equilibration part (31–35 min): 0.0 min 5% B; 25 min 30% B; 25.5 min 95% B; 30.9 min 95% B; 31 min 5% B; 35 min 5% B. A standard-flow electrospray was used to couple the UHPLC system with a triple quadrupole mass spectrometer (AJS 6495A, Agilent, CA). Electrospray source operated in positive ion mode (capillary voltage 3.5 kV; gas flow rate 11 L/min at 130 °C; sheath gas pressure 25 PSI at 400 °C; nozzle voltage 500 V). We monitored 98 transitions per the dynamic SRM mode analysis, with 2 min window scheduled around peptide experimental RT. SRM signature transitions were equivalent for proteotypic peptide and corresponding SIL internal standard, i.e., a single SRM quantifier transition and 2–4 additional qualifier SRM transitions were acquired (Table S4).

### Protein assay validation and reproducibility

We extracted proteins from all 134 meconium and feces swabs using the protocol and pooled protein extracts (70 µL) from all individual samples. We prepared the dilution series adding SIL-TCT or SIL peptides (10 µL) into the pooled extract and applied trypsin digestion protocol and UHPLC-SRM. We determined the linearity range, the limit of detection (LOD), the limit of quantification (LOQ), inter-day and intra-day precision of multiplex SRM protein assay. A dilution series consisted of up to 9 concentration levels, measured at 3–6 technical replicates. A separate calibration curve was used for low abundant ECP and EDN proteins. For interday and intraday reproducibility, protein extracts were prepared at seven concentration levels (i.e., 0.1; 0.5; 1; 2; 4; 6, and 8 g/l) in triplicate, processed, and analyzed in three consecutive days (n = 6 each day).

### Data analysis

SRM assays were refined using Skyline (Version 19.1.0.193; MacCoss Lab, Uni of Washington, WA). We acquired integrated peak areas in MassHunter (Agilent, CA), performed statistical analysis for method validation using Excel (Microsoft Office Professional Plus, 2013), and performed statistical analyses in GraphPad Prism. LOD and LOQ were determined from the matrix-matched calibration curve. The standard deviation (SD) at the lowest concentration level with a coefficient of variation (CV) < 20% (n = 6) was used for calculation, e.g., 3*SD divided by SLOPE (for LOD) and 10*SD divided by SLOPE (for LOQ). The coefficient of determinations was R^2^ > 0.99, determined using Spearman correlation was performed in GraphPad Prism. Mann–Whitney *U* test was used for comparison of two groups in GraphPad Prism (Version 8.3.0).

## Results and discussion

### Proteolysis reproducibility and peptide yields

The varied amount of neonatal feces collected on a swab is challenging to measure, which hampers the absolute protein quantification. We used the correlation between feces fresh weight (FW) and the total protein content measured by BCA to determine the amount of stool collected on a swab (Figure S1 and Table S3). We tested the reproducibility of trypsin digestion after 1, 3, 5, 17, 20, and 24 h of incubation using QC sample protein extracts with added SIL-TCT peptides. Peptide concentrations and CV (n = 3) were determined (Table S5). Surrogate peptides were not detected in the control sample (0 h, no trypsin added). The surrogate peptides' yields were optimal within 5 h of incubation; CV < 16.7% (Figure S2).

### Validation of multiplex protein assay

Protein assays for A1AT-1, IGHA1, IGHA2, IGHA1 + 2, ECP, EDN, MPO, CAL1, and CAL2 were validated using matrix-matched calibration curves for each SIL-TCT peptide standard in terms of linearity of response, R^2^, LOD, and LOQ. Protein assays were linear within the range of two orders of magnitude at minimum, typically from 0.04 to 14.77 µg/mg of the total protein content. All R^2^ were > 0.99. LODs and LOQs of target proteins in the sample matrix ranged from 0.003 to 0.04 µg/mg and from 0.009 to 0.122 µg/mg of total protein content, respectively (Table 2).

We established the intra- and inter-day precision in QC samples within a single day (n = 6) and three consecutive days (n = 6). The intraday median CV precision was 9.8%; all CV values < 17.8% (Table S6). The interday median CV precision was 14.1%, all CV values < 16.2%, except for peptide TPLTATLSK (IGHA1) with the CV of 28.9% (Table S7). The peptide VVLEGGIDPILR (MPO) concentration in the QC sample was below LOQ (29.9% CV).

### Robustness of multiplex protein assays

Meconium or feces FW collected on a swab ranged between 25 and 150 mg, corresponding to 0.97–10.34 µg/µL of extracted total protein concentration. The extraction efficiency or kinetics of trypsin digestion and overall protein assay accuracy may be affected by variable protein concentration in extracts. We tested the robustness of A1AT-1, IGHA1, IGHA2, IGHA1 + 2, ECP, EDN, MPO, CAL1, and CAL2 quantification in QC sample stool extracts varied in total protein concentration (i.e., 0.1; 0.5; 1; 2; 4; 6 and 8 µg/µL, range 5–400 µg/ 50µL of extract). The assay was robust as all protein concentrations were determined within CV < 20.6% (Table S8 and Figure S3), except for low abundant EDN (CV 25.1%).

### Immune markers in meconium and feces

**Figure 2:**
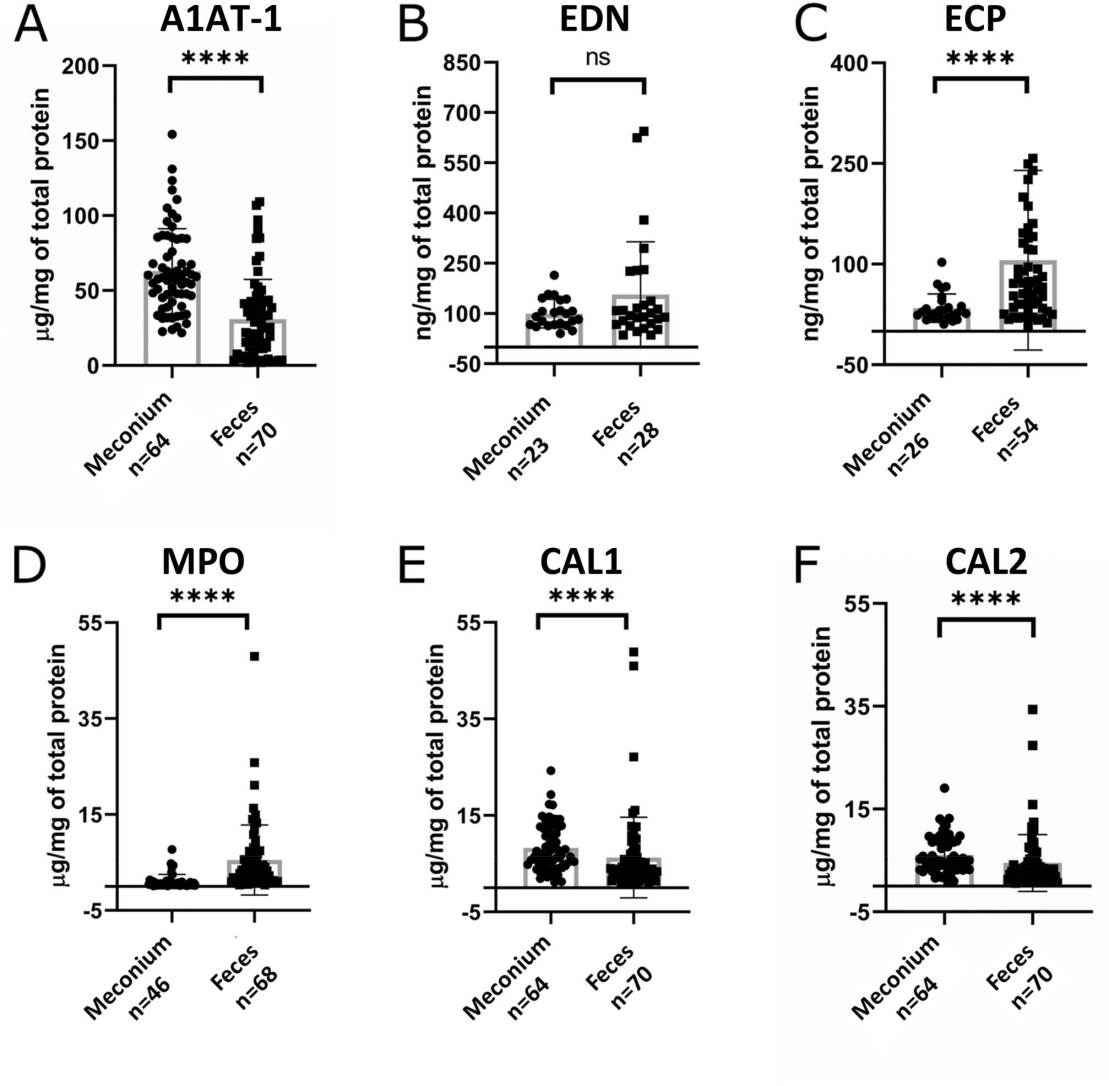
Levels of immune protein markers in meconium and feces: (**A**) A1AT-1, (**B**) EDN, (**C**) ECP, (**D**) MPO, (**E**) CAL1, (**F**) CAL2. Mann–Whitney *U* test ****P < 0.0001.

Meconium initially excreted by neonates transitions into a first feces around the second day after birth due to breastfeeding^32^. We quantified fecal IGHA1 and IGHA2 levels introduced from breast milk to capture the transition and differentiate meconium and first feces samples. We classified 64 samples as meconium (IGHA < 10 µg/mg of total protein) and 70 samples as the first feces (IGHA > 10 µg/mg of total protein). We compared levels of immune markers in meconium (n = 64) and feces (n = 70) samples with the Mann–Whitney *U* test (Fig. 2). We found significantly higher A1AT-1, CAL1, CAL2, ECP, and MPO levels in meconium relative to feces (P < 0.0001). Higher levels of blood plasma-derived A1AT-1 (Fig. 2A) in meconium indicate permeability of immature intestinal barrier for plasma proteins and blood cells^39^. The breast milk consumption induces the tightening of intestinal barrier function and reduces blood proteins' leakage into the feces^32^. The initial colonization by gut microbiota, enhanced by breast milk intake, causes the release of MPO from neutrophils attacking bacteria^17^. Presumably, a reason for significantly higher MPO levels in feces compared to meconium (P < 0.0001).

**Figure 3:**
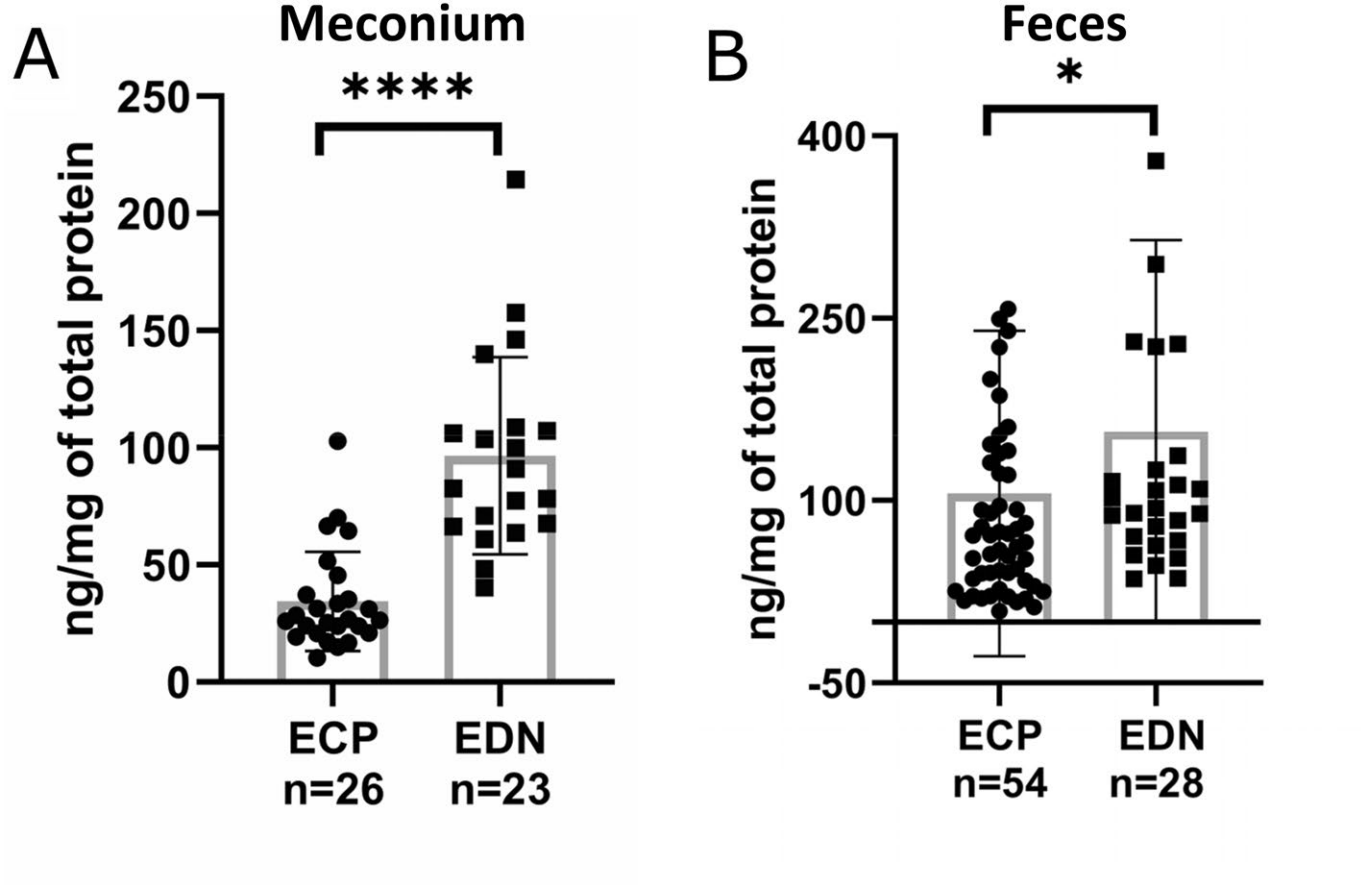
ECP and EDN levels in meconium and feces. (**A**) meconium (n = 41) and (**B**) feces (n = 70), Mann–Whitney *U* test, *P < 0.05, ****P < 0.0001.

The breast milk intake probably contributes to allergic sensitization^40^. ECP and EDN from eosinophils play a role in the host defense against a pathogen^41^. ECP and EDN have an antiviral activity to single strained RNA viruses^42^. ECP has marked antibacterial activity and toxicity for helminth parasites. On the other hand, EDN activates dendric cells to express inflammatory chemokines and cytokines, activates the TLR2- MyD88 signal pathway, and enhances Th2 immune responses^43^. The presence of ECP and EDN released from blood-born eosinophils in the intestinal lumen indicates endogenous allergic inflammation^22,31^. While we determined significantly increased ECP levels in the first feces relative to meconium, the increase of EDN was not significant (Fig. 2). Perhaps, a consequence of the permeable intestine on the first day of life, allowing for a massive non-specific infiltration of meconium by other white blood cells expressing EDN (e.g., basophils and neutrophils)^44^. In consecutive days of life, the intestine permeability decreases, allowing only eosinophils to infiltrate and contribute to fecal EDN. We cross-compared ECP and EDN levels in meconium and feces to assess other white blood cells' potential contribution to meconium EDN (Fig. 3). If ECP and EDN in meconium and feces are derived only from eosinophils, the EDN/ECP ratio in meconium and feces is expected similar. The EDN/ECP ratio in meconium was substantially higher than in the first feces suggesting a contribution from other white blood cells to the total meconium EDN level. However, the different EDN/ECP ratios may also reflect changes in eosinophil protein expression after the first day of life.

**Figure 4:**
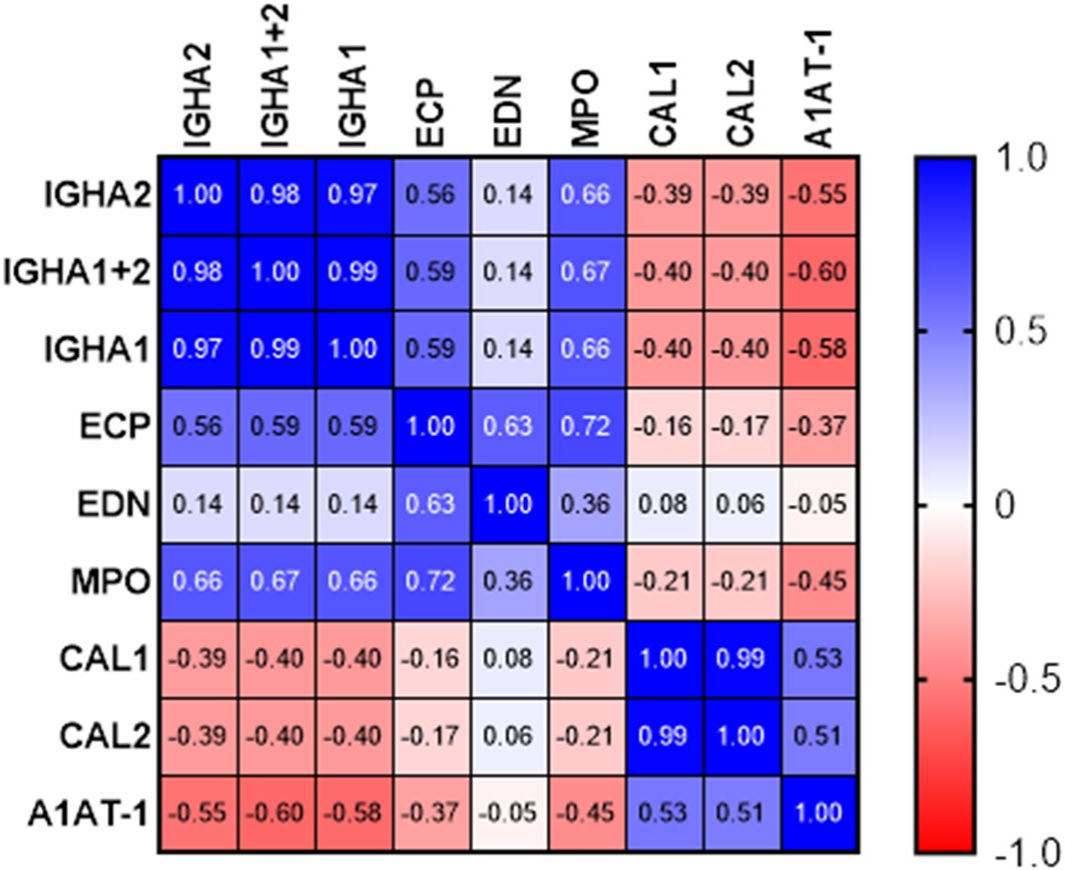
Correlation matrix plot for IGHA, A1AT-1, IGHA1, IGHA2, IGHA1 + 2, ECP, EDN, MPO, CAL1, and CAL2 levels in neonatal stool samples (n = 134).

A1AT-1, IGHA1, IGHA2, IGHA1 + 2, ECP, EDN, MPO, CAL1, and CAL2 levels (Table S2) in meconium and feces (n = 134), sorted by decreasing concentration of IGHA were cross-correlated using the Spearman correlation factor (Fig. 4). The excretory IGHA1 + 2 in feces and meconium negatively correlated with A1AT-1 (Spearman correlation factor of − 0.6), as previously reported^32^. Li et al. demonstrated decreasing calprotectin levels in neonatal stool between 1 to 18 months^45^. Indeed, we detected a negative correlation of IGHA1 + 2 with both CAL1 and CAL2 (Spearman correlation factor of − 0.4). MPO and ECP moderately correlate with IGHA1 + 2 (Spearman correlation factor of 0.67 and 0.59, respectively). MPO was associated with ECP (Spearman correlation factor of 0.72) and ECP with EDN (Spearman correlation factor of 0.63).

### Birth mode influence on immune protein levels in meconium and feces

Meconium and feces samples were from neonates delivered vaginally (VD) and via cesarean section (CS). VD reportedly fosters the eubiotic bacterial colonization of the intestinal lumen^46^. Conversely, skin commensals, opportunistic pathogens, or nosocomial bacteria primarily colonize CS neonates' intestinal lumen. CAL1 and CAL2 levels indicate the neutrophil type of inflammation in response to a pathogen's presence in the intestinal lumen^17^. The comparison of CAL1 and CAL2 levels in the feces of VD (n = 58) and CS (n = 12) neonates revealed significantly higher CAL1 levels (*P* value 0.0439) in CS neonates (Figure S4). CAL1 and CAL2 are components of the calprotectin dimer complex, but the CAL2 level was not significantly different between CS and VD neonates (*P* value 0.0622). However, fecal calprotectin levels were highly varied in neonates, and the sample size was limited. Meconium CAL1 and CAL2 in CS and VD neonates were not significantly different (Figure S4). Increased fecal calprotectin is a potential marker of dysbiosis and low-grade inflammation of the gastrointestinal tract in CS neonates.

## Conclusions

In summary, we developed and validated a multiplex mass spectrometry-based protein assay for absolute quantification of adaptive immunity effectors (i.e., IGHA1, IGHA2) and immune protein markers (i.e., A1AT, ECP, EDN, MPO, CAL1, CAL2) in neonatal meconium and feces swabs. While circulating immune protein markers are routinely quantified in blood/serum, neonatal excretory levels are not established. We determined the stool (fresh weight) collected on a swab by measuring protein concentration in protein extracts. Information on the precise amount collected on a stool swab is required for absolute protein quantification. Breast milk-derived IGHA2 differentiates meconium from feces to study the influence of breastfeeding on intestinal barrier function maturation. Absolute quantification of multiple immune protein markers characterizes the type of inflammation in the intestinal lumen. We found significantly higher fecal calprotectin levels in neonates delivered via Cesarean section relative to vaginal delivery birth. The finding may indicate a low-grade inflammation in response to microbial dysbiosis in CS neonates.

## Supplementary Information

Supplementary Information.

## Data Availability

The mass spectrometry data were deposited to the PANORAMA Repository (https://panoramaweb.org/U%20of%20Masaryk%20-%20RECETOX/Vidova_MultiMec_Assay/project-begin.view?pageId=Raw%20Data).
