# Supplementary material for: Simultaneous quantitative profiling of clinically relevant immune markers in neonatal stool swabs to reveal inflammation
Source: Sci Rep. 2021 May 13;11:10222. doi: 10.1038/s41598-021-89384-0 (PMC8119937; doi:10.1038/s41598-021-89384-0)
Supplement: Supplementary file 1 — Supplementary Information. [file 41598_2021_89384_MOESM1_ESM.docx]

Supplementary information for the manuscript:

Simultaneous quantitative profiling of clinically relevant immune markers in neonatal stool swabs to reveal inflammation

*Veronika Vidova, Eliska Benesova, Jana Klanova, Vojtech Thon, Zdenek Spacil**

Masaryk University, Faculty of Science, RECETOX Centre, Brno, Czech Republic

Address correspondence to Dr. Zdenek Spacil, Masaryk University, Faculty of Science, Kamenice 753/5, pavilion D29/418, 625 00 Brno, Czech Republic. Tel. (+420) 549 49 7989; e-mail: [spacil@recetox.muni.cz](mailto:spacil@recetox.muni.cz) or spacil@u.washington.edu

**Figures and Tables Legend**

**Figure S1.** The correlation between the fresh weight and total protein concentration in the stool sample extract.

**Figure S2.** Optimization of incubation time for trypsin digestion. Concentrations of peptides after 1, 3, 5, 17, 20, and 24 hours of incubation. The concentration of IGHA1 and IGHA2 peptides: SAVQGPPER (■), DASGATFTWTPSSGK (▲), and TPLTATLSK (●). B) The concentration of A1AT-1 peptide AVLTIDEK (▼). C) The concentration of CAL1 and CAL2 peptides: DLQNFLK (□), LGHPDTLNQGEFK (○), and ALNSIIDVYHK (♦). D) The concentration of ECP and EDN peptides: DPPQYPVVPVHLDR (◊) and NQNTFLR (+). E) The concentration of MPO peptide VVLEGGIDPILR (x).

**Figure S3.** The robustness of protein assay in samples containing various amounts of fresh stool. Protein concentrations were determined in QC sample extracts with total protein content ranging from 0.1 to 8.0 μg/μl (equivalent to 10.3 mg to 115.2 mg stool fresh weight): (A) IGHA1 and IGHA2 peptides: SAVQGPPER (■), DASGATFTWTPSSGK (▲) and TPLTATLSK (●). A1AT-1 peptide AVLTIDEK (▼). The concentration of CAL1 and CAL2 peptides: DLQNFLK (□), LGHPDTLNQGEFK (○), and ALNSIIDVYHK (♦). (B) The concentration of EDN and ECP peptides: DPPQYPVVPVHLDR (◊) and NQNTFLR (+) and concentration of MPO peptide: VVLEGGIDPILR (x). Concentrations of EDN in 0.1 to 1 μg/μl protein extracts were below LOQ (not shown). Concentrations of ECP and MPO in 0.1 and 0.5 μg/μl protein extracts were below LOQ (not shown).

**Figure S4.** The comparison of CAL1 (A) and CAL2 (B) levels in the stool of neonates delivered vaginally (VD, n=58) and via cesarean section (CS, n=12) neonates revealed significantly higher CAL1 levels (P-value 0.0439) in CS neonates. Mann-Whitney *U* test * P<0.05.

**Table S1.** Demographic and clinical characteristics of all study subjects and information on sample collection.

**Table S2.** The total protein content and absolute concentrations of inflammatory proteins (A1AT-1, IGHA1, IGHA2, IGHA1+2, ECP, EDN, MPO, CAL1, and CAL2) in meconium and first feces from 134 neonates. Samples from Cesarean section neonates(n=20) are highlighted in bold font.

**Table S3.** Correlation between stool sample fresh weight and total protein concentration in the protein extract. Various stool amounts were accurately weighed (25, 50, 75, 100, 125, 150 mg) on a swab in triplicate. The protein content in protein extracts using the BCA assay was determined and the correlation between the stool sample fresh weight and total protein content established.

**Table S4.** Transition list of surrogate peptides generated in SRMAtlas. Product ions used for peptide quantification are highlighted in bold font.

**Table S5.** Optimization of trypsin digestion time. We determined peptide concentration after 1, 3, 5, 17, 20, and 24 hours of incubation.

**Table S6.** Intraday precision of absolute quantification of A1AT-1, IGHA1, IGHA2, IGHA1+2, ECP, EDN, MPO, CAL1, and CAL2 in QC samples (n=6).

**Table S7.** Interday precision of absolute quantification of A1AT-1, IGHA1, IGHA2, IGHA1+2, ECP, EDN, MPO, CAL1, and CAL2 in QC samples in three consecutive days (n=6 per day).

**Table S8**. Robustness of protein assay. Absolute protein quantification in various amounts of fresh weight (0.1 to 8.0 µg/µl of total protein concentration in extract) in stool samples.

**Figure S1**

**

**

**Figure S2**

**
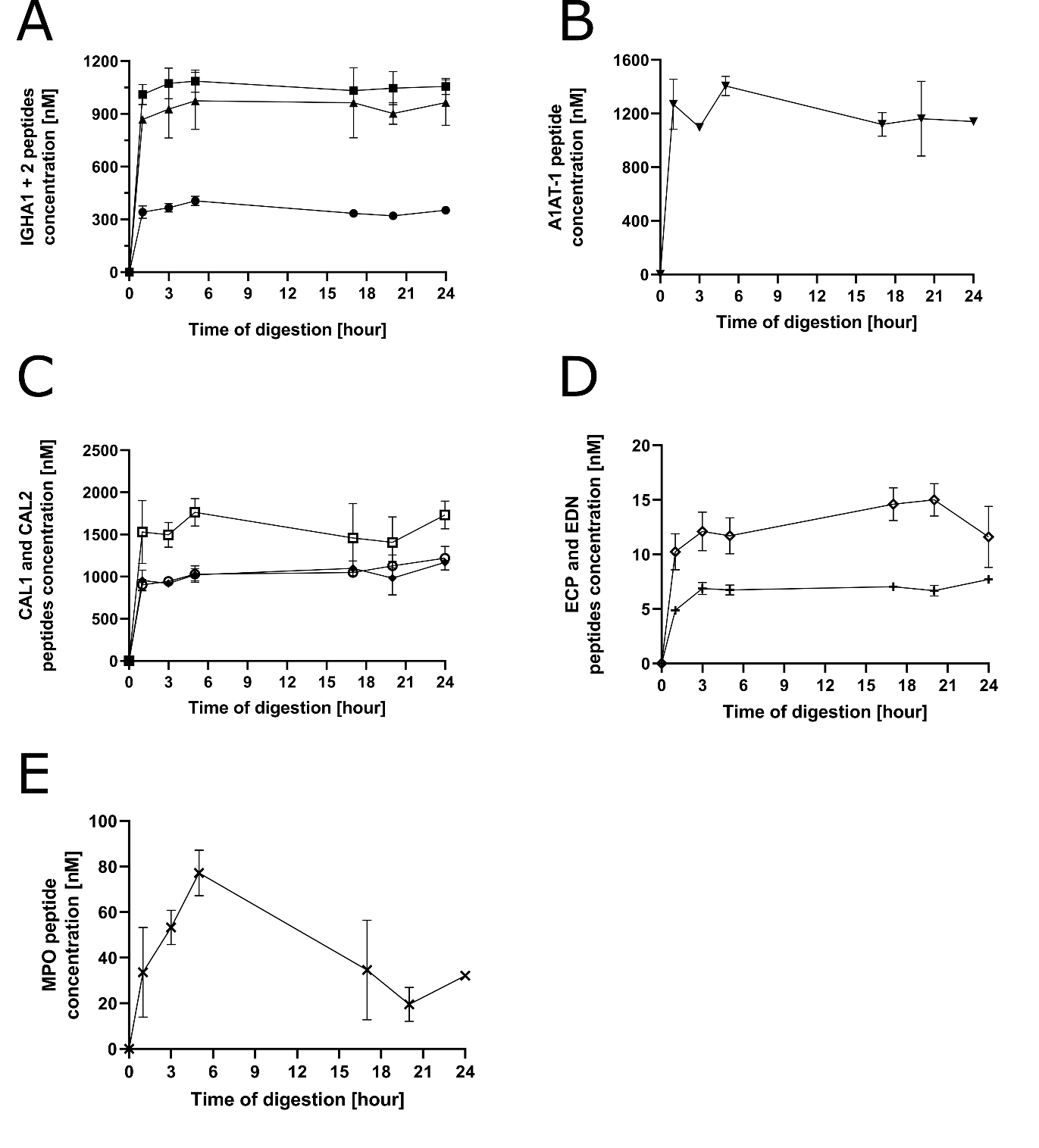
**

**Figure S3**

**
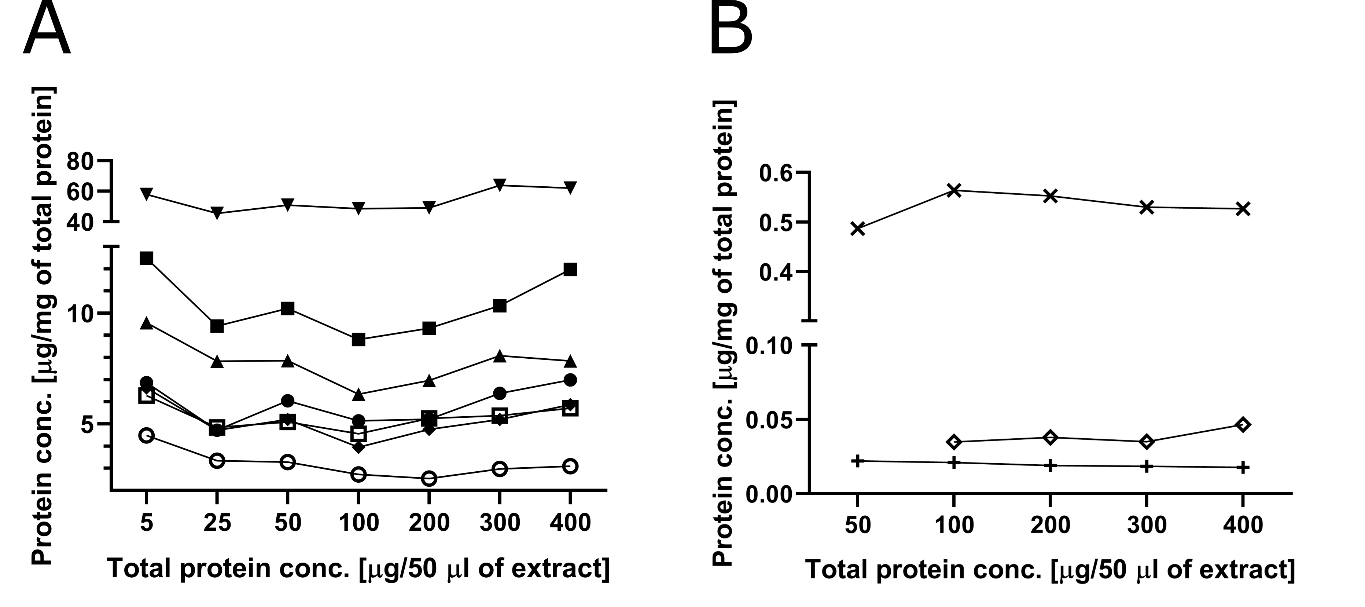
**

**Figure S4**

**
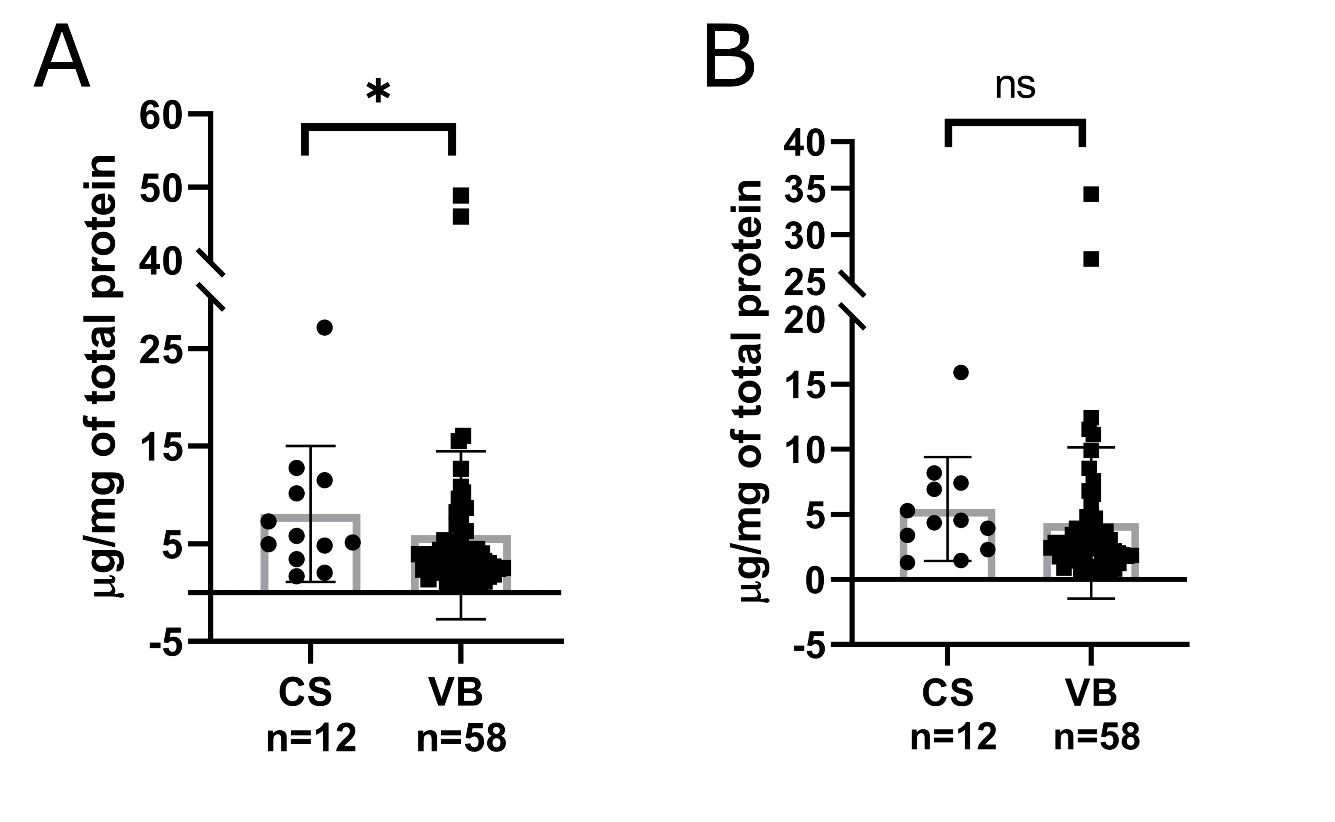
**

**CS VD CS VD**

**Table S1**

| **Family ID** | **Gestation age** | **Mode of delivery** | **Sex** | **Length (cm)** | **Weight (g)** | **Apgar score** | **Visual assessment** | **Number of days from birth to sample collection** | **Sample collection (date/time)** | **Sample transport (date/time)** | **Sample arrival to lab (date/time)** | **Sample placed into a freezer (date/time)** |
| --- | --- | --- | --- | --- | --- | --- | --- | --- | --- | --- | --- | --- |
| 21 | 40+2 | vaginal | M | 51 | 3600 | [9, 10, 10] | meconium | 3 | 02.11.2016 11:05 | 02.11.2016 11:10 | 02.11.2016 11:38 | 02.11.2016 11:39:19 |
| 25 | 39+6 | vaginal | M | 50 | 3240 | [10, 10, 10] | meconium | 2 | 25.11.2016 11:05 | 25.11.2016 11:20 | 25.11.2016 11:45 | 25.11.2016 11:47:04 |
| 27 | 40+4 | vaginal | F | 52 | 3410 | [6, 9, 10] | meconium | 3 | 11.11.2016 07:35 | 11.11.2016 07:45 | 11.11.2016 07:45 | 11.11.2016 08:59:39 |
| 28 | 40+4 | vaginal | M | 49 | 3240 | [9, 9, 10] | meconium | 2 | 01.12.2016 08:35 | 01.12.2016 08:45 | 01.12.2016 08:55 | 01.12.2016 09:14:46 |
| 31 | 41+0 | vaginal | F | 50 | 3760 | [8, 9, 9] | stool | 3 | 21.11.2016 07:15 | 21.11.2016 07:50 | 21.11.2016 08:00 | 21.11.2016 09:13:58 |
| 32 | 39+6 | Caesarean | F | 47 | 3220 | [10, 10, 10] | stool | 3 | 25.11.2016 08:40 | 25.11.2016 08:50 | 25.11.2016 09:20 | 25.11.2016 09:25:18 |
| 36 | 39+3 | vaginal | M | 51 | 3230 | [3, 7, 9] | stool | 3 | 25.11.2016 08:25 | 25.11.2016 08:50 | 25.11.2016 09:15 | 25.11.2016 09:25:29 |
| 37 | 40+1 | vaginal | M | 52 | 3630 | [10, 10, 10] | stool | 2 | 15.09.2016 10:50 | 15.09.2016 10:55 | 15.09.2016 12:00 | 15.09.2016 12:01:31 |
| 40 | 40+1 | vaginal | F | 49 | 3320 | [10, 10, 10] | stool | 2 | 14.09.2016 10:25 | 14.09.2016 10:32 | 14.09.2016 10:40 | 14.09.2016 12:36:07 |
| 50 | 39+1 | vaginal | M | 50 | 3240 | [10, 10, 10] | stool | 2 | 18.10.2016 08:30 | 18.10.2016 08:35 | 18.10.2016 09:10 | 18.10.2016 09:49:08 |
| 55 | 40+0 | vaginal | F | 49 | 3820 | [10, 10, 10] | meconium | 2 | 09.01.2017 11:10 | 09.01.2017 11:30 | 09.01.2017 12:00 | 09.01.2017 13:48:52 |
| 56 | 41+3 | Caesarean | M | 49 | 3250 | [9, 9, 10] | meconium | 2 | 25.01.2017 10:50 | 25.01.2017 10:55 | 25.01.2017 11:00 | 25.01.2017 12:07:15 |
| 57 | 39+3 | Caesarean | M | 46 | 2490 | [5, 8, 8] | stool | 3 | 23.01.2017 09:40 | 23.01.2017 09:50 | 23.01.2017 10:00 | 23.01.2017 10:13:35 |
| 63 | 40+2 | vaginal | F | 49 | 3320 | [10, 10, 10] | meconium | 1 | 08.12.2016 08:35 | 08.12.2016 08:45 | 08.12.2016 09:05 | 08.12.2016 09:05:18 |
| 65 | 41+0 | vaginal | F | 49 | 3690 | [9, 10, 10] | meconium | 2 | 13.12.2016 11:20 | 13.12.2016 11:25 | 13.12.2016 11:30 | 13.12.2016 11:33:35 |
| 66 | 40+2 | vaginal | M | 50 | 3360 | [10, 10, 10] | meconium | 3 | 09.12.2016 09:15 | 09.12.2016 09:20 | 09.12.2016 09:30 | 09.12.2016 10:30:05 |
| 67 | 40+5 | Caesarean | M | 51 | 3900 | [7, 7, 8] | stool | 3 | 07.12.2016 15:05 | 07.12.2016 15:15 | 07.12.2016 15:20 | 07.12.2016 15:24:05 |
| 68 | 40+4 | vaginal | M | 54 | 3910 | [10, 10, 10] | meconium | 1 | 09.12.2016 11:20 | 09.12.2016 11:30 | 09.12.2016 11:30 | 09.12.2016 12:27:55 |
| 70 | 40+5 | vaginal | F | 50 | 3600 | [9, 10, 10] | stool | 3 | 13.12.2016 06:45 | 13.12.2016 07:00 | 13.12.2016 07:10 | 13.12.2016 07:11:37 |
| 71 | 39+5 | Caesarean | F | 51 | 3790 | [9, 10, 10] | meconium | 1 | 02.12.2016 10:05 | 02.12.2016 10:15 | 02.12.2016 10:20 | 02.12.2016 10:23:20 |
| 72 | 40+0 | vaginal | M | 52 | 3800 | [10, 10, 10] | meconium | 3 | 06.12.2016 07:45 | 06.12.2016 08:05 | 06.12.2016 08:10 | 06.12.2016 08:19:57 |
| 77 | 38+5 | vaginal | F | 48 | 2750 | [9, 9, 9] | meconium | 3 | 26.01.2017 07:40 | 26.01.2017 08:10 | 26.01.2017 09:00 | 26.01.2017 09:28:08 |
| 79 | 39+3 | vaginal | M | 52 | 3950 | [9, 9, 10] | stool | 2 | 01.02.2017 08:50 | 01.02.2017 09:05 | 01.02.2017 09:15 | 01.02.2017 09:17:34 |
| 82 | 40+0 | vaginal | M | 48 | 3200 | [9, 10, 10] | meconium | 2 | 06.02.2017 10:05 | 06.02.2017 10:30 | 06.02.2017 10:40 | 06.02.2017 10:52:14 |
| 90 | 39+3 | vaginal | F | 52 | 3520 | [9, 10, 10] | stool | 1 | 15.02.2017 14:10 | 15.02.2017 14:20 | 15.02.2017 14:45 | 15.02.2017 14:46:07 |
| 95 | 38+6 | vaginal | M | 49 | 3030 | [9, 10, 10] | meconium | 1 | 21.02.2017 12:10 | 21.02.2017 12:15 | 21.02.2017 12:30 | 21.02.2017 12:33:33 |
| 116 | 39+5 | vaginal | M | 53 | 4100 | [10, 10, 10] | meconium | 2 | 25.04.2017 09:20 | 25.04.2017 09:25 | 25.04.2017 09:40 | 25.04.2017 10:14:51 |
| 117 | 39+4 | vaginal | F | 48 | 3040 | [10, 10, 10] | meconium | 1 | 21.04.2017 10:10 | 21.04.2017 10:20 | 21.04.2017 10:30 | 21.04.2017 12:48:31 |
| 121 | 40+4 | vaginal | M | 51 | 3730 | [9, 10, 10] | meconium | 3 | 02.05.2017 14:20 | 02.05.2017 14:25 | 02.05.2017 14:35 | 02.05.2017 14:52:06 |
| 122 | 40+3 | vaginal | M | 60 | 3390 | [9, 10, 10] | meconium | 1 | 15.05.2017 14:30 | 15.05.2017 14:35 | 15.05.2017 14:45 | 15.05.2017 14:50:28 |
| 124 | 40+3 | vaginal | M | 51 | 3770 | [9, 10, 10] | meconium | 3 | 18.05.2017 07:35 | 18.05.2017 07:45 | 18.05.2017 08:00 | 18.05.2017 08:47:02 |
| 125 | 39+2 | vaginal | M | 48 | 2960 | [10, 10, 10] | stool | 3 | 11.05.2017 08:15 | 11.05.2017 08:30 | 11.05.2017 08:30 | 11.05.2017 08:40:02 |
| 127 | 39+4 | vaginal | F | 49 | 3210 | [10, 10, 10] | meconium | 1 | 19.05.2017 11:50 | 19.05.2017 12:15 | 19.05.2017 12:45 | 19.05.2017 13:40:58 |
| 128 | 40+2 | vaginal | M | 49 | 3370 | [9, 10, 10] | meconium | 2 | 10.05.2017 10:45 | 10.05.2017 11:05 | 10.05.2017 11:15 | 10.05.2017 12:26:45 |
| 130 | 40+5 | vaginal | F | 49 | 3200 | [9, 10, 10] | meconium | 3 | 31.05.2017 08:00 | 31.05.2017 08:05 | 31.05.2017 08:10 | 31.05.2017 09:41:18 |
| 134 | 39+4 | vaginal | M | 50 | 3360 | [9, 10, 10] | meconium | 1 | 24.02.2017 14:55 | 24.02.2017 13:05 | 24.02.2017 15:00 | 24.02.2017 15:16:33 |
| 140 | 39+5 | vaginal | F | 50 | 3650 | [8, 10, 10] | stool | 3 | 27.02.2017 10:35 | 27.02.2017 10:45 | 27.02.2017 12:00 | 27.02.2017 12:34:26 |
| 141 | 38+6 | vaginal | M | 49 | 3260 | [9, 10, 10] | meconium | 2 | 16.05.2017 07:45 | 16.05.2017 08:05 | 16.05.2017 08:20 | 16.05.2017 08:54:41 |
| 142 | 40+2 | vaginal | M | 52 | 3540 | [10, 10, 10] | meconium | 2 | 29.05.2017 09:45 | 29.05.2017 09:50 | 29.05.2017 10:20 | 29.05.2017 13:29:32 |
| 143 | 39+6 | vaginal | F | 51 | 3550 | [9, 9, 9] | stool | 3 | 27.02.2017 11:20 | 27.02.2017 11:30 | 27.02.2017 12:00 | 27.02.2017 12:34:17 |
| 144 | 40+2 | vaginal | M | 51 | 3590 | [8, 9, 9] | stool | 3 | 07.03.2017 14:00 | 07.03.2017 14:05 | 07.03.2017 15:00 | 07.03.2017 14:55:55 |
| 145 | 40+2 | vaginal | F | 50 | 3700 | [9, 9, 10] | stool | 3 | 27.02.2017 07:40 | 27.02.2017 08:05 | 27.02.2017 08:30 | 27.02.2017 08:37:00 |
| 146 | 39+5 | vaginal | M | 51 | 2900 | [9, 10, 10] | meconium | 2 | 15.05.2017 07:45 | 15.05.2017 07:55 | 15.05.2017 08:10 | 15.05.2017 08:30:52 |
| 147 | 39+5 | vaginal | M | 53 | 3540 | [8, 10, 10] | meconium | 2 | 30.05.2017 07:25 | 30.05.2017 07:35 | 30.05.2017 08:00 | 30.05.2017 10:46:40 |
| 148 | 39+3 | Caesarean | F | 50 | 2880 | [10, 10, 10] | stool | 2 | 01.03.2017 10:00 | 01.03.2017 10:10 | 01.03.2017 10:30 | 01.03.2017 10:34:41 |
| 150 | 39+1 | vaginal | M | 51 | 3740 | [9, 10, 10] | meconium | 2 | 07.03.2017 12:50 | 07.03.2017 13:00 | 07.03.2017 14:00 | 07.03.2017 14:00:49 |
| 153 | 40+6 | vaginal | M | 51 | 4180 | [5, 8, 9] | meconium | 2 | 16.03.2017 07:50 | 16.03.2017 07:55 | 16.03.2017 08:10 | 16.03.2017 10:52:06 |
| 154 | 39+0 | Caesarean | F | 50 | 3000 | [10, 10, 10] | stool | 3 | 13.03.2017 11:10 | 13.03.2017 11:25 | 13.03.2017 11:50 | 13.03.2017 12:01:57 |
| 158 | 40+4 | vaginal | F | 51 | 3460 | [10, 10, 10] | meconium | 1 | 17.03.2017 14:25 | 17.03.2017 14:35 | 17.03.2017 14:45 | 17.03.2017 14:46:46 |
| 159 | 40+1 | vaginal | F | 50 | 3120 | [10, 10, 10] | stool | 3 | 27.03.2017 10:40 | 27.03.2017 10:50 | 27.03.2017 11:00 | 27.03.2017 11:18:14 |
| 160 | 40+0 | Caesarean | M | 49 | 3780 | [9, 10, 10] | stool | 3 | 27.03.2017 11:50 | 27.03.2017 12:10 | 27.03.2017 12:20 | 27.03.2017 12:41:58 |
| 161 | 39+3 | vaginal | M | 51 | 2870 | [9, 10, 10] | meconium | 2 | 24.03.2017 11:20 | 24.03.2017 11:50 | 24.03.2017 12:30 | 24.03.2017 13:26:25 |
| 163 | 40+3 | vaginal | M | 52 | 3550 | [10, 10, 10] | stool | 3 | 27.03.2017 10:25 | 27.03.2017 10:50 | 27.03.2017 11:00 | 27.03.2017 11:18:25 |
| 167 | 40+2 | vaginal | F | 47 | 2810 | [9, 10, 10] | meconium | 1 | 02.06.2017 07:20 | 02.06.2017 07:35 | 02.06.2017 11:00 | 02.06.2017 12:52:55 |
| 171 | 40+2 | vaginal | F | 51 | 3380 | [10, 10, 10] | meconium | 2 | 30.03.2017 08:20 | 30.03.2017 08:30 | 30.03.2017 08:50 | 30.03.2017 09:16:18 |
| 172 | 39+6 | vaginal | F | 52 | 3340 | [9, 10, 10] | meconium | 1 | 31.03.2017 10:45 | 31.03.2017 10:55 | 31.03.2017 11:00 | 31.03.2017 12:17:05 |
| 173 | 40+4 | vaginal | M | 55 | 4190 | [9, 10, 10] | meconium | 3 | 05.04.2017 07:40 | 05.04.2017 07:50 | 05.04.2017 08:00 | 05.04.2017 08:56:52 |
| 176 | 40+5 | vaginal | F | 49 | 2670 | [10, 10, 10] | meconium | 1 | 04.04.2017 09:55 | 04.04.2017 10:15 | 04.04.2017 10:40 | 04.04.2017 10:51:14 |
| 177 | 40+1 | vaginal | M | 52 | 3420 | [9, 10, 10] | meconium | 1 | 19.05.2017 12:00 | 19.05.2017 12:15 | 19.05.2017 12:45 | 19.05.2017 13:40:49 |
| 180 | 41+1 | vaginal | F | 55 | 4290 | [9, 9, 10] | stool | 3 | 10.04.2017 07:50 | 10.04.2017 08:05 | 10.04.2017 09:00 | 10.04.2017 10:56:32 |
| 181 | 40+6 | vaginal | F | 50 | 3220 | [9, 9, 10] | meconium | 2 | 06.06.2017 08:05 | 06.06.2017 08:25 | 06.06.2017 08:30 | 06.06.2017 09:24:46 |
| 182 | 40+3 | Caesarean | M | 53 | 4900 | [10, 10, 10] | meconium | 2 | 25.05.2017 13:45 | 25.05.2017 13:50 | 25.05.2017 14:00 | 25.05.2017 14:25:18 |
| 183 | 40+5 | vaginal | M | 49 | 3300 | [10, 10, 10] | meconium | 3 | 05.06.2017 09:55 | 05.06.2017 10:05 | 05.06.2017 10:15 | 05.06.2017 10:23:00 |
| 184 | 38+5 | vaginal | F | 49 | 2750 | [9, 9, 10] | meconium | 2 | 06.06.2017 07:45 | 06.06.2017 08:25 | 06.06.2017 08:30 | 06.06.2017 09:24:36 |
| 185 | 40+6 | vaginal | F | 52 | 3860 | [9, 9, 9] | stool | 2 | 08.06.2017 07:35 | 08.06.2017 07:45 | 08.06.2017 08:00 | 08.06.2017 10:22:11 |
| 186 | 40+3 | vaginal | M | 54 | 3740 | [7, 9, 9] | meconium | 1 | 15.06.2017 13:00 | 15.06.2017 13:10 | 15.06.2017 13:40 | 15.06.2017 13:43:22 |
| 188 | 40+3 | vaginal | M | 50 | 3030 | [10, 10, 10] | stool | 2 | 07.06.2017 14:05 | 07.06.2017 14:10 | 07.06.2017 14:15 | 07.06.2017 14:30:40 |
| 190 | 39+4 | vaginal | M | 50 | 3380 | [9, 9, 9] | stool | 3 | 08.06.2017 10:10 | 08.06.2017 10:35 | 08.06.2017 10:45 | 08.06.2017 10:59:12 |
| 192 | 38+5 | Caesarean | M | 49 | 4070 | [9, 9, 9] | meconium | 2 | 08.06.2017 09:35 | 08.06.2017 09:50 | 08.06.2017 10:00 | 08.06.2017 10:22:31 |
| 204 | 40+5 | vaginal | F | 51 | 3300 | [9, 10, 10] | meconium | 1 | 26.06.2017 13:45 | 26.06.2017 13:40 | 26.06.2017 14:00 | 26.06.2017 14:58:38 |
| 206 | 40+1 | vaginal | M | 49 | 3280 | [9, 9, 9] | meconium | 1 | 21.06.2017 09:40 | 21.06.2017 09:45 | 21.06.2017 10:00 | 21.06.2017 09:53:16 |
| 207 | 40+0 | vaginal | F | 48 | 2840 | [9, 9, 10] | meconium | 1 | 26.06.2017 07:20 | 26.06.2017 07:30 | 26.06.2017 07:50 | 26.06.2017 08:02:36 |
| 242 | 40+6 | vaginal | M | 51 | 3570 | [8, 9, 10] | meconium/stool | 3 | 08.08.2017 10:15 | 08.08.2017 10:25 | 08.08.2017 11:00 | 08.08.2017 11:36:32 |
| 243 | 39+2 | vaginal | M | 50 | 3150 | [10, 10, 10] | meconium | 1 | 24.07.2017 11:15 | 24.07.2017 11:35 | 24.07.2017 11:45 | 24.07.2017 12:52:08 |
| 244 | 41+2 | vaginal | M | 51 | 3580 | [5, 8, 9] | 0 | 1 | 31.07.2017 09:45 | 31.07.2017 09:55 | 31.07.2017 12:30 | 31.07.2017 12:44:38 |
| 245 | 40+5 | vaginal | F | 50 | 3120 | [5, 7, 9] | meconium | 2 | 31.07.2017 12:45 | 31.07.2017 12:50 | 31.07.2017 13:30 | 31.07.2017 13:35:39 |
| 249 | 40+2 | vaginal | M | 52 | 4350 | [8, 9, 10] | stool | 3 | 02.02.2017 12:30 | 02.02.2017 12:35 | 02.02.2017 12:50 | 02.02.2017 12:51:55 |
| 253 | 41+0 | vaginal | M | 50 | 3420 | [9, 10, 10] | meconium | 1 | 14.08.2017 11:40 | 14.08.2017 12:20 | 14.08.2017 12:30 | 14.08.2017 12:43:37 |
| 254 | 40+1 | vaginal | M | 52 | 3290 | [5, 7, 8] | meconium | 2 | 03.08.2017 10:35 | 03.08.2017 10:40 | 03.08.2017 11:00 | 03.08.2017 11:09:50 |
| 256 | 40+5 | Caesarean | M | 52 | 3700 | [9, 10, 10] | stool | 3 | 07.08.2017 07:25 | 07.08.2017 07:45 | 07.08.2017 08:10 | 07.08.2017 08:20:12 |
| 258 | 39+4 | vaginal | F | 50 | 4140 | [9, 10, 10] | stool | 3 | 14.08.2017 11:30 | 14.08.2017 12:20 | 14.08.2017 12:30 | 14.08.2017 12:43:25 |
| 259 | 40+5 | vaginal | F | 52 | 3440 | [8, 10, 10] | meconium | 1 | 09.08.2017 07:20 | 09.08.2017 07:40 | 09.08.2017 08:30 | 09.08.2017 09:58:06 |
| 260 | 41+1 | vaginal | F | 52 | 3930 | [9, 9, 10] | stool | 3 | 22.08.2017 09:50 | 22.08.2017 10:35 | 22.08.2017 10:45 | 22.08.2017 11:05:31 |
| 261 | 40+5 | vaginal | M | 51 | 3790 | [10, 10, 10] | meconium | 2 | 18.08.2017 07:25 | 18.08.2017 07:35 | 18.08.2017 08:20 | 18.08.2017 08:27:58 |
| 263 | 40+3 | vaginal | F | 52 | 3760 | [10, 10, 10] | stool | 3 | 04.09.2017 08:50 | 04.09.2017 09:05 | 04.09.2017 09:10 | 08.09.2017 11:02:24 |
| 264 | 40+4 | vaginal | F | 52 | 3640 | [10, 10, 10] | stool | 2 | 23.08.2017 07:20 | 23.08.2017 07:45 | 23.08.2017 07:50 | 23.08.2017 07:57:26 |
| 266 | 40+0 | vaginal | M | 50 | 2850 | [9, 9, 10] | stool | 3 | 21.08.2017 11:05 | 21.08.2017 11:15 | 21.08.2017 11:20 | 21.08.2017 11:42:52 |
| 267 | 39+2 | Caesarean | M | 50 | 3810 | [1, 6, 8] | meconium | 2 | 24.08.2017 11:25 | 24.08.2017 11:40 | 24.08.2017 11:50 | 24.08.2017 12:16:02 |
| 268 | 38+6 | vaginal | M | 50 | 3530 | [9, 10, 10] | meconium | 2 | 21.08.2017 09:15 | 21.08.2017 09:50 | 21.08.2017 10:00 | 21.08.2017 10:18:29 |
| 269 | 38+6 | vaginal | F | 50 | 3430 | [10, 10, 10] | stool | 0 | 22.08.2017 07:30 | 22.08.2017 07:50 | 22.08.2017 08:00 | 22.08.2017 08:53:36 |
| 270 | 40+0 | vaginal | F | 47 | 3230 | [10, 10, 10] | meconium | 2 | 23.08.2017 07:30 | 23.08.2017 07:45 | 23.08.2017 07:45 | 23.08.2017 07:57:15 |
| 273 | 40+4 | vaginal | M | 52 | 3810 | [9, 10, 10] | stool | 3 | 04.09.2017 14:05 | 04.09.2017 14:15 | 04.09.2017 14:20 | 08.09.2017 11:02:52 |
| 274 | 39+2 | vaginal | F | 50 | 3250 | [10, 10, 10] | stool | 3 | 04.09.2017 08:55 | 04.09.2017 09:05 | 04.09.2017 09:15 | 08.09.2017 11:02:44 |
| 275 | 39+2 | vaginal | M | 50 | 3900 | [9, 10, 10] | meconium | 2 | 05.09.2017 13:30 | 05.09.2017 13:35 | 05.09.2017 13:50 | 08.09.2017 11:02:38 |
| 276 | 39+2 | Caesarean | M | 49 | 3130 | [9, 9, 10] | meconium | 3 | 28.08.2017 10:50 | 28.08.2017 11:00 | 28.08.2017 11:15 | 28.08.2017 12:15:50 |
| 279 | 41+3 | vaginal | F | 50 | 3700 | [9, 10, 10] | stool | 3 | 19.09.2017 09:55 | 19.09.2017 10:00 | 19.09.2017 10:10 | 19.09.2017 10:15:14 |
| 284 | 38+6 | Caesarean | F | 50 | 3950 | [10, 10, 10] | stool | 3 | 26.09.2017 07:40 | 26.09.2017 08:00 | 26.09.2017 09:00 | 26.09.2017 09:06:17 |
| 290 | 39+3 | vaginal | F | 51 | 3510 | [10, 10, 10] | stool | 3 | 29.09.2017 07:30 | 29.09.2017 07:45 | 29.09.2017 08:00 | 29.09.2017 08:07:48 |
| 291 | 40+5 | vaginal | M | 50 | 3750 | [9, 9, 9] | meconium | 1 | 09.10.2017 12:50 | 09.10.2017 13:00 | 09.10.2017 13:15 | 09.10.2017 14:13:53 |
| 295 | 39+6 | vaginal | M | 50 | 3200 | [9, 10, 10] | meconium | 0 | 05.10.2017 09:55 | 05.10.2017 10:00 | 05.10.2017 10:05 | 05.10.2017 10:18:19 |
| 296 | 39+3 | vaginal | F | 54 | 3820 | [9, 10, 10] | meconium | 1 | 03.10.2017 14:20 | 03.10.2017 14:40 | 03.10.2017 14:50 | 03.10.2017 15:02:50 |
| 297 | 40+1 | vaginal | F | 51 | 4010 | [9, 10, 10] | stool | 3 | 09.10.2017 09:55 | 09.10.2017 10:50 | 09.10.2017 11:00 | 09.10.2017 11:20:25 |
| 298 | 41+0 | vaginal | M | 51 | 3730 | [9, 10, 10] | meconium | 1 | 24.10.2017 08:40 | 24.10.2017 08:55 | 24.10.2017 09:00 | 24.10.2017 09:06:10 |
| 300 | 40+2 | vaginal | M | 50 | 3300 | [8, 9, 10] | stool | 3 | 16.10.2017 09:40 | 16.10.2017 09:45 | 16.10.2017 09:50 | 16.10.2017 09:54:13 |
| 303 | 40+1 | vaginal | M | 51 | 3590 | [10, 10, 10] | stool | 3 | 09.10.2017 11:00 | 09.10.2017 11:10 | 09.10.2017 11:15 | 09.10.2017 11:20:36 |
| 309 | 39+1 | Caesarean | M | 47 | 2960 | [1, 0, 0] | meconium/stool | 2 | 17.10.2017 10:35 | 17.10.2017 10:45 | 17.10.2017 11:00 | 17.10.2017 11:16:31 |
| 311 | 40+2 | vaginal | M | 50 | 3000 | [10, 10, 10] | meconium/stool | 1 | 25.10.2017 14:55 | 25.10.2017 15:05 | 25.10.2017 15:30 | 25.10.2017 15:45:53 |
| 312 | 40+0 | Caesarean | F | 47 | 2930 | [9, 10, 10] | stool | 3 | 17.10.2017 08:15 | 17.10.2017 08:30 | 17.10.2017 08:30 | 17.10.2017 09:30:48 |
| 313 | 39+4 | Caesarean | M | 47 | 3300 | [9, 10, 10] | meconium | 2 | 23.10.2017 14:40 | 23.10.2017 14:45 | 23.10.2017 14:55 | 23.10.2017 14:57:29 |
| 320 | 39+0 | vaginal | M | 47 | 2470 | [9, 10, 10] | meconium | 3 | 25.10.2017 08:40 | 25.10.2017 08:50 | 25.10.2017 09:00 | 25.10.2017 10:02:51 |
| 322 | 41+3 | vaginal | M | 51 | 4190 | [10, 10, 10] | stool | 1 | 25.10.2017 13:10 | 25.10.2017 13:45 | 25.10.2017 14:00 | 25.10.2017 14:07:29 |
| 324 | 41+0 | vaginal | F | 53 | 3550 | [8, 8, 9] | stool | 3 | 13.11.2017 08:10 | 14.11.2017 08:15 | 14.11.2017 08:30 | 14.11.2017 09:13:00 |
| 325 | 39+5 | vaginal | F | 50 | 3730 | [9, 10, 10] | meconium | 1 | 26.10.2017 11:40 | 27.10.2017 08:15 | 27.10.2017 08:30 | 27.10.2017 08:42:46 |
| 326 | 39+3 | Caesarean | M | 50 | 2960 | [9, 9, 9] | meconium/stool | 3 | 09.11.2017 07:50 | 10.11.2017 08:15 | 10.11.2017 08:30 | 10.11.2017 08:45:52 |
| 327 | 41+1 | vaginal | M | 52 | 3170 | [8, 10, 10] | meconium | 0 | 15.11.2017 13:15 | 0 | 15.11.2017 14:15 | 15.11.2017 14:17:20 |
| 329 | 40+3 | vaginal | F | 51 | 3680 | [9, 10, 10] | meconium | 2 | 31.10.2017 08:45 | 01.11.2017 08:15 | 01.11.2017 08:45 | 01.11.2017 09:21:26 |
| 330 | 40+4 | vaginal | M | 50 | 4130 | [10, 10, 10] | stool | 3 | 03.11.2017 07:45 | 06.11.2017 08:15 | 06.11.2017 08:50 | 06.11.2017 09:01:20 |
| 331 | 40+4 | vaginal | F | 51 | 4110 | [9, 9, 10] | meconium | 1 | 07.11.2017 13:55 | 0 | 07.11.2017 14:20 | 07.11.2017 14:26:03 |
| 333 | 39+2 | vaginal | M | 48 | 3180 | [9, 10, 10] | meconium | 1 | 10.11.2017 14:10 | 0 | 10.11.2017 14:35 | 10.11.2017 14:45:24 |
| 334 | 40+0 | vaginal | F | 51 | 3820 | [9, 9, 9] | meconium | 2 | 10.11.2017 07:50 | 0 | 10.11.2017 08:30 | 10.11.2017 08:46:18 |
| 336 | 40+6 | vaginal | F | 53 | 4450 | [9, 10, 10] | meconium/stool | 3 | 16.11.2017 10:50 | 20.11.2017 08:15 | 20.11.2017 08:30 | 20.11.2017 09:16:18 |
| 337 | 39+0 | vaginal | M | 48 | 3230 | [9, 9, 9] | meconium | 2 | 09.11.2017 11:25 | 0 | 09.11.2017 11:45 | 09.11.2017 12:29:52 |
| 338 | 38+2 | Caesarean | M | 49 | 3230 | [9, 10, 10] | meconium/stool | 3 | 15.11.2017 09:55 | 16.11.2017 08:15 | 16.11.2017 08:30 | 16.11.2017 09:13:16 |
| 339 | 41+1 | Caesarean | F | 53 | 3930 | [10, 10, 10] | stool | 3 | 14.11.2017 07:40 | 15.11.2017 08:15 | 15.11.2017 09:15 | 15.11.2017 09:19:32 |
| 340 | 40+2 | vaginal | M | 51 | 4000 | [10, 10, 10] | meconium/stool | 1 | 10.11.2017 14:00 | 0 | 10.11.2017 14:35 | 10.11.2017 14:45:29 |
| 344 | 40+6 | vaginal | M | 53 | 4130 | [7, 9, 10] | meconium/stool | 2 | 20.11.2017 11:05 | 0 | 20.11.2017 12:00 | 20.11.2017 12:18:49 |
| P3-1 | 40+1 | vaginal | F | 52 | 3620 | [9, 9, 10] | meconium | 2 | 05.08.2016 8:30 | 0 | 0 | 05.08.2016 8:40 |
| P3-2 | 39+4 | vaginal | M | 49 | 3450 | [7, 8, 9] | meconium | 2 | 08.08.2016 11:35 | 0 | 0 | 08.08.2016 11:40 |
| P3-3 | 40+6 | vaginal | F | 52 | 3690 | [10, 10, 10] | meconium/stool | 2 | 24.08.2016 10:00 | 0 | 0 | 24.08.2016 10:20 |
| P3-4 | 40+2 | vaginal | M | 50 | 3380 | [9, 9, 9] | stool | 2 | 17.08.2016 10:30 | 0 | 0 | 17.08.2016 10:40 |
| P3-5 | 40+4 | vaginal | M | 54 | 4560 | [9, 10, 10] | meconium | 2 | 16.08.2016 7:40 | 0 | 0 | 16.08.2016 7:50 |
| P3-6 | 39+6 | vaginal | M | 50 | 3090 | [9, 10, 10] | meconium | 2 | 26.08.2016 11:45 | 0 | 0 | 26.08.2016 12:00 |
| P3-7 | 39+5 | vaginal | F | 49 | 2980 | [9, 10, 10] | meconium/stool | 2 | 26.08.2016 8:45 | 0 | 0 | 26.08.2016 10:00 |
| P3-8 | 40+1 | vaginal | M | 52 | 3430 | [10, 10, 10] | meconium | 2 | 29.08.2016 11:10 | 0 | 0 | 29.08.2016 14:00 |

**Table S2**

| **Sample ID** | **Total protein concentration [µg/µl]** | **Absolute protein concentration relative to the total protein content** | | | | | | | | |
| --- | --- | --- | --- | --- | --- | --- | --- | --- | --- | --- |
|  |  | **A1AT-1 [µg/mg]** | **IGHA2 [µg/mg]** | **IGHA1+2 [µg/mg]** | **IGHA1 [µg/mg]** | **ECP [ng/mg]** | **EDN [ng/mg]** | **MPO [µg/mg]** | **CAL1 [µg/mg]** | **CAL2 [µg/mg]** |
| 21 | 2.48 | 67.38 | <LOQ | <LOQ | <LOQ | 64.43 | 106.17 | 3.98 | 14.31 | 10.32 |
| 25 | 4.10 | 33.40 | <LOQ | <LOQ | <LOQ | 26.69 | 60.86 | 0.38 | 1.93 | 1.55 |
| 27 | 2.42 | 84.99 | <LOQ | <LOQ | <LOQ | 21.03 | <LOQ | 1.17 | 14.21 | 8.66 |
| 28 | 3.86 | 54.15 | <LOQ | <LOQ | <LOQ | 10.41 | <LOQ | 0.49 | 4.82 | 3.93 |
| 31 | 0.36 | 52.34 | 18.10 | 22.73 | 16.20 | <LOQ | <LOQ | 3.26 | 45.96 | 27.40 |
| **32** | **0.84** | **15.49** | **86.12** | **98.81** | **69.26** | **121.32** | **<LOQ** | **4.95** | **27.14** | **15.90** |
| 36 | 1.10 | 12.22 | 97.24 | 108.41 | 111.90 | 51.90 | <LOQ | 2.16 | 3.28 | 2.39 |
| 37 | 3.08 | 154.29 | 0.21 | 0.36 | 0.11 | <LOQ | 64.23 | 0.20 | 4.43 | 3.17 |
| 40 | 3.11 | 62.84 | <LOQ | <LOQ | <LOQ | <LOQ | <LOQ | 0.61 | 8.27 | 5.25 |
| 50 | 2.02 | 12.76 | 86.24 | 148.10 | 116.61 | 92.54 | 78.86 | 7.49 | 2.31 | 1.85 |
| 55 | 0.11 | 97.08 | 6.86 | 14.99 | 9.64 | <LOQ | <LOQ | 3.88 | 48.85 | 34.40 |
| **56** | **3.47** | **42.41** | **<LOQ** | **<LOQ** | **0.03** | **16.87** | **<LOQ** | **2.98** | **3.83** | **3.18** |
| **57** | **1.12** | **1.88** | **39.73** | **54.33** | **51.58** | **42.45** | **<LOQ** | **2.62** | **1.67** | **1.30** |
| 63 | 0.88 | 101.33 | 4.96 | 7.73 | 3.78 | <LOQ | <LOQ | 7.73 | 17.31 | 11.79 |
| 65 | 4.27 | 32.96 | 11.02 | 10.19 | 3.29 | 9.09 | 52.70 | 0.76 | 8.21 | 12.41 |
| 66 | 4.72 | 72.74 | 74.74 | 86.19 | 38.56 | 40.21 | 55.30 | 2.27 | 2.10 | 1.47 |
| **67** | **2.52** | **21.80** | **<LOQ** | **0.14** | **<LOQ** | **25.26** | **70.97** | **1.38** | **1.26** | **0.97** |
| 68 | 1.96 | 58.54 | 0.40 | 0.64 | 0.29 | 37.22 | 82.52 | <LOQ | 5.01 | 4.50 |
| 70 | 3.26 | 14.16 | 64.74 | 163.37 | 109.46 | 43.88 | <LOQ | 4.56 | 3.00 | 2.42 |
| **71** | **3.14** | **47.76** | **<LOQ** | **<LOQ** | **<LOQ** | **<LOQ** | **99.83** | **0.87** | **9.03** | **7.30** |
| 72 | 3.46 | 21.87 | 27.97 | 88.59 | 69.63 | 146.18 | 89.87 | 11.75 | 2.29 | 1.73 |
| 77 | 1.36 | 98.41 | 2.13 | 3.64 | 2.21 | <LOQ | <LOQ | 4.41 | 11.75 | 9.29 |
| 79 | 2.52 | 33.21 | 100.65 | 97.70 | 43.29 | 21.47 | <LOQ | 0.31 | 2.04 | 1.91 |
| 82 | 2.97 | 75.95 | <LOQ | 0.13 | 0.08 | <LOQ | <LOQ | 0.26 | 3.25 | 2.81 |
| 90 | 3.38 | 52.00 | <LOQ | <LOQ | <LOQ | <LOQ | <LOQ | 0.13 | 12.27 | 9.81 |
| 95 | 2.39 | 38.90 | <LOQ | <LOQ | <LOQ | 23.88 | <LOQ | <LOQ | 6.42 | 4.58 |
| 116 | 1.73 | 96.05 | 0.34 | 0.41 | 0.18 | <LOQ | <LOQ | 0.27 | 2.66 | 1.95 |
| 117 | 4.12 | 60.73 | <LOQ | <LOQ | 0.04 | <LOQ | 40.35 | <LOQ | 19.34 | 13.17 |
| 121 | 3.82 | 47.98 | <LOQ | <LOQ | <LOQ | <LOQ | <LOQ | 1.13 | 7.06 | 5.28 |
| 122 | 0.69 | 84.51 | <LOQ | <LOQ | <LOQ | <LOQ | <LOQ | 0.63 | 14.06 | 10.06 |
| 124 | 1.18 | 4.61 | 209.00 | 265.24 | 140.29 | <LOQ | <LOQ | 2.33 | 2.54 | 1.76 |
| 125 | 0.70 | 90.93 | 18.00 | 23.34 | 13.21 | 257.75 | 624.66 | 16.31 | 15.51 | 11.14 |
| 127 | 1.56 | 50.99 | <LOQ | <LOQ | <LOQ | <LOQ | <LOQ | 0.99 | 24.25 | 19.05 |
| 128 | 1.94 | 31.79 | <LOQ | <LOQ | <LOQ | <LOQ | <LOQ | <LOQ | 7.76 | 5.11 |
| 130 | 3.56 | 43.62 | 62.16 | 104.18 | 57.48 | 25.35 | <LOQ | 1.10 | 7.72 | 6.55 |
| 134 | 1.56 | 85.49 | <LOQ | <LOQ | <LOQ | <LOQ | <LOQ | <LOQ | 5.33 | 4.31 |
| 140 | 2.34 | 7.66 | 42.87 | 101.81 | 76.61 | 239.79 | 228.88 | 6.44 | 1.42 | 1.08 |
| 141 | 3.44 | 46.44 | 13.80 | 15.59 | 4.55 | 19.38 | <LOQ | 5.64 | 4.28 | 3.43 |
| 142 | 3.23 | 27.75 | 140.02 | 156.15 | 52.75 | 89.51 | 113.06 | 4.54 | 0.90 | 0.65 |
| 143 | 3.54 | 22.53 | 2.50 | 2.26 | 0.79 | 66.56 | 157.70 | 4.77 | 2.23 | 1.71 |
| 144 | 2.08 | 3.65 | 54.29 | 47.52 | 19.90 | 21.22 | <LOQ | 0.99 | 1.14 | 0.88 |
| 145 | 1.32 | 2.08 | 68.68 | 116.84 | 95.76 | 199.94 | 137.03 | 25.84 | 4.01 | 3.21 |
| 146 | 3.66 | 59.31 | <LOQ | <LOQ | <LOQ | <LOQ | <LOQ | <LOQ | 2.78 | 2.09 |
| 147 | 2.42 | 117.01 | <LOQ | <LOQ | <LOQ | 51.51 | <LOQ | <LOQ | 2.90 | 1.91 |
| **148** | **2.88** | **44.21** | **13.54** | **20.97** | **18.07** | **81.62** | **125.16** | **7.38** | **10.17** | **6.92** |
| 150 | 2.84 | 48.43 | 5.43 | 6.98 | 2.30 | 17.20 | 107.29 | 0.49 | 6.17 | 4.26 |
| 153 | 2.12 | 106.95 | 95.27 | 80.47 | 23.31 | 95.72 | 115.76 | 6.77 | 12.66 | 9.92 |
| **154** | **0.71** | **84.74** | **19.06** | **20.89** | **6.17** | **<LOQ** | **<LOQ** | **1.93** | **11.51** | **7.41** |
| 158 | 2.62 | 54.22 | <LOQ | 0.10 | <LOQ | <LOQ | <LOQ | 0.27 | 12.68 | 9.38 |
| 159 | 1.78 | 25.38 | 8.03 | 13.61 | 9.30 | 76.66 | 108.28 | 1.40 | 9.62 | 6.78 |
| **160** | **1.88** | **16.40** | **132.57** | **165.29** | **65.57** | **29.69** | **<LOQ** | **2.20** | **2.01** | **1.45** |
| 161 | 4.22 | 60.19 | 1.97 | 2.87 | 1.68 | 45.61 | 77.32 | 0.63 | 6.80 | 4.32 |
| 163 | 0.55 | 3.37 | 86.84 | 288.99 | 235.81 | 78.58 | <LOQ | 14.04 | 5.95 | 4.71 |
| 167 | 2.63 | 109.28 | 14.76 | 19.47 | 21.12 | 35.55 | 89.33 | 1.02 | 16.07 | 11.51 |
| 171 | 3.68 | 86.95 | <LOQ | <LOQ | <LOQ | <LOQ | <LOQ | 0.38 | 7.85 | 5.04 |
| 172 | 2.10 | 105.01 | <LOQ | <LOQ | <LOQ | 19.22 | <LOQ | <LOQ | 14.22 | 10.96 |
| 173 | 3.50 | 25.41 | 46.02 | 67.25 | 39.26 | 71.31 | 46.72 | 4.26 | 4.08 | 2.83 |
| 176 | 3.94 | 92.72 | <LOQ | <LOQ | <LOQ | 28.48 | 63.56 | 0.66 | 6.21 | 4.16 |
| 177 | 1.55 | 32.67 | <LOQ | <LOQ | 0.10 | <LOQ | <LOQ | 0.57 | 9.48 | 7.87 |
| 180 | 5.30 | 11.53 | 492.96 | 108.23 | 83.00 | 40.50 | 87.71 | 14.95 | 0.73 | 0.56 |
| 181 | 3.90 | 42.36 | 13.00 | 19.97 | 11.66 | 19.25 | 70.48 | 0.40 | 5.22 | 3.92 |
| **182** | **2.23** | **56.83** | **<LOQ** | **<LOQ** | **<LOQ** | **<LOQ** | **<LOQ** | **<LOQ** | **5.59** | **4.06** |
| 183 | 1.28 | 54.35 | 30.24 | 50.72 | 28.10 | <LOQ | <LOQ | 0.85 | 3.98 | 2.99 |
| 184 | 2.69 | 72.49 | 0.17 | 0.23 | 0.06 | 14.96 | <LOQ | 0.61 | 12.51 | 8.77 |
| 185 | 2.34 | 33.64 | 35.32 | 44.81 | 27.95 | <LOQ | <LOQ | <LOQ | 10.83 | 7.56 |
| 186 | 2.29 | 131.03 | <LOQ | 0.20 | 0.17 | 31.29 | 143.09 | 0.44 | 7.97 | 5.86 |
| 188 | 1.52 | 16.90 | 93.46 | 208.56 | 160.43 | <LOQ | <LOQ | 1.30 | 1.86 | 1.41 |
| 190 | 0.84 | 3.44 | 29.42 | 98.74 | 78.92 | <LOQ | <LOQ | 1.15 | 0.94 | 0.68 |
| **192** | **1.52** | **50.28** | **25.08** | **29.08** | **16.33** | **52.43** | **<LOQ** | **2.71** | **5.11** | **4.36** |
| 204 | 2.87 | 54.93 | <LOQ | 0.13 | 0.12 | 20.67 | 108.66 | 0.18 | 12.89 | 8.36 |
| 206 | 2.68 | 56.91 | <LOQ | <LOQ | <LOQ | 26.33 | <LOQ | 0.22 | 6.64 | 4.52 |
| 207 | 1.82 | 86.80 | <LOQ | <LOQ | <LOQ | <LOQ | <LOQ | <LOQ | 12.99 | 8.56 |
| 242 | 2.02 | 85.02 | 8.79 | 13.40 | 6.82 | <LOQ | <LOQ | 0.61 | 3.23 | 2.20 |
| 243 | 2.62 | 67.92 | 7.48 | 9.16 | 6.30 | <LOQ | <LOQ | 0.80 | 4.83 | 3.70 |
| 244 | 3.99 | 39.41 | <LOQ | <LOQ | <LOQ | <LOQ | <LOQ | 0.11 | 8.47 | 5.41 |
| 245 | 5.73 | 47.71 | 0.17 | 0.13 | 0.02 | 102.76 | 146.13 | 2.51 | 6.11 | 4.95 |
| 249 | 0.14 | 110.81 | <LOQ | 2.56 | <LOQ | <LOQ | <LOQ | <LOQ | 14.72 | 12.53 |
| 253 | 2.06 | 45.25 | <LOQ | <LOQ | <LOQ | <LOQ | <LOQ | <LOQ | 1.19 | 0.81 |
| 254 | 3.67 | 65.33 | 1.41 | 1.21 | 0.22 | <LOQ | <LOQ | 0.73 | 4.03 | 2.88 |
| **256** | **0.32** | **38.62** | **99.92** | **127.29** | **47.27** | **<LOQ** | **<LOQ** | **<LOQ** | **4.94** | **3.92** |
| 258 | 1.68 | 2.83 | 62.35 | 134.51 | 104.17 | 71.84 | <LOQ | 5.26 | 3.68 | 2.79 |
| 259 | 5.68 | 21.16 | 32.20 | 40.83 | 14.60 | 21.61 | <LOQ | 3.50 | 3.65 | 2.82 |
| 260 | 3.44 | 36.77 | 189.46 | 288.12 | 118.82 | 131.36 | 102.20 | 9.73 | 4.31 | 3.07 |
| 261 | 4.00 | 37.75 | 2.42 | 4.00 | 1.78 | 70.01 | 214.56 | 0.82 | 5.41 | 4.48 |
| 263 | 0.26 | 3.40 | 77.00 | 196.18 | 176.74 | <LOQ | <LOQ | 5.38 | 3.94 | 3.69 |
| 264 | 3.05 | 33.55 | 34.37 | 39.31 | 9.67 | 16.45 | 67.15 | 1.24 | 2.53 | 1.80 |
| 266 | 1.63 | 40.27 | 138.43 | 149.83 | 97.97 | 138.86 | <LOQ | 4.34 | 2.45 | 1.66 |
| **267** | **2.00** | **84.24** | **4.40** | **3.69** | **0.34** | **31.21** | **139.87** | **0.78** | **3.80** | **3.24** |
| 268 | 1.05 | 34.00 | 4.88 | 4.57 | 0.36 | <LOQ | <LOQ | 1.38 | 6.78 | 5.84 |
| 269 | 1.68 | 4.42 | 72.84 | 83.42 | 70.01 | 249.36 | 230.85 | 13.29 | 2.70 | 2.04 |
| 270 | 2.06 | 35.53 | 32.19 | 56.03 | 38.88 | 61.96 | 93.82 | 2.26 | 8.66 | 5.89 |
| 273 | 1.24 | 28.90 | 81.54 | 83.16 | 50.99 | 34.05 | <LOQ | 3.42 | 2.36 | 1.52 |
| 274 | 1.32 | 6.28 | 99.43 | 229.71 | 175.99 | 59.16 | <LOQ | 6.59 | 3.37 | 2.60 |
| 275 | 4.78 | 38.74 | 38.18 | 85.96 | 58.53 | <LOQ | <LOQ | 2.70 | 3.28 | 2.43 |
| **276** | **1.40** | **17.93** | **79.96** | **200.75** | **148.69** | **74.49** | **<LOQ** | **3.93** | **3.44** | **2.30** |
| 279 | 2.79 | 41.22 | 33.96 | 37.94 | 17.73 | 735.02 | 644.31 | 47.95 | 3.56 | 3.32 |
| **284** | **0.22** | **62.75** | **<LOQ** | **<LOQ** | **<LOQ** | **<LOQ** | **<LOQ** | **<LOQ** | **17.16** | **13.07** |
| 290 | 3.89 | 12.57 | 19.74 | 24.55 | 11.48 | 92.17 | 226.47 | 10.83 | 1.93 | 1.37 |
| 291 | 2.10 | 60.32 | <LOQ | <LOQ | 0.05 | <LOQ | <LOQ | 0.59 | 5.06 | 3.59 |
| 295 | 1.96 | 85.53 | <LOQ | <LOQ | <LOQ | 33.40 | 78.14 | <LOQ | 4.51 | 3.13 |
| 296 | 2.62 | 46.61 | 5.80 | 3.78 | 0.91 | 25.86 | 103.72 | 0.41 | 14.85 | 9.67 |
| 297 | 2.15 | 8.81 | 84.55 | 193.85 | 121.36 | 655.06 | 379.39 | 13.41 | 3.38 | 2.36 |
| 298 | 2.30 | 59.60 | <LOQ | <LOQ | <LOQ | <LOQ | <LOQ | 0.20 | 4.34 | 3.40 |
| 300 | 0.86 | 1.86 | 62.21 | 42.00 | 72.50 | <LOQ | <LOQ | 1.05 | 1.61 | 1.21 |
| 303 | 0.78 | 15.13 | 92.93 | 161.15 | 96.00 | 122.25 | <LOQ | 9.23 | 4.46 | 3.32 |
| **309** | **1.29** | **19.40** | **39.43** | **61.69** | **39.87** | **154.30** | **<LOQ** | **2.71** | **5.80** | **4.53** |
| 311 | 4.19 | 23.94 | <LOQ | <LOQ | <LOQ | <LOQ | 48.25 | 0.25 | 3.42 | 2.94 |
| **312** | **0.27** | **2.81** | **9.72** | **32.44** | **24.74** | **160.67** | **<LOQ** | **8.11** | **7.31** | **5.29** |
| **313** | **2.02** | **123.39** | **0.68** | **2.13** | **1.50** | **35.26** | **155.85** | **0.56** | **7.61** | **5.27** |
| 320 | 0.39 | 20.01 | 84.68 | 126.92 | 70.42 | 186.25 | <LOQ | 21.10 | 10.27 | 8.54 |
| 322 | 2.54 | 55.44 | <LOQ | <LOQ | <LOQ | <LOQ | <LOQ | 0.30 | 5.79 | 4.72 |
| 324 | 2.94 | 15.75 | 120.15 | 236.17 | 170.01 | <LOQ | <LOQ | 1.52 | 1.10 | 0.77 |
| 325 | 1.03 | 33.28 | <LOQ | 0.24 | 0.16 | <LOQ | <LOQ | <LOQ | 10.72 | 8.59 |
| **326** | **2.15** | **42.43** | **18.70** | **32.63** | **18.27** | **25.71** | **83.71** | **1.07** | **4.81** | **3.38** |
| 327 | 0.62 | 67.84 | <LOQ | 0.70 | 0.36 | <LOQ | <LOQ | <LOQ | 10.26 | 7.66 |
| 329 | 2.80 | 57.53 | <LOQ | <LOQ | <LOQ | <LOQ | <LOQ | 0.37 | 13.32 | 9.78 |
| 330 | 1.18 | 62.82 | 8.89 | 10.30 | 3.36 | 55.75 | <LOQ | 4.12 | 5.22 | 3.84 |
| 331 | 3.56 | 42.67 | 44.24 | 54.07 | 21.02 | <LOQ | <LOQ | 0.48 | 6.98 | 4.80 |
| 333 | 1.33 | 31.34 | <LOQ | <LOQ | <LOQ | <LOQ | <LOQ | <LOQ | 4.00 | 2.84 |
| 334 | 3.43 | 25.80 | <LOQ | 0.09 | 0.04 | <LOQ | 66.33 | 0.23 | 6.52 | 4.82 |
| 336 | 3.30 | 27.95 | 0.13 | 0.11 | 0.03 | <LOQ | <LOQ | 0.71 | 12.81 | 8.97 |
| 337 | 1.03 | 4.31 | 66.47 | 131.62 | 79.32 | 72.68 | 294.33 | 4.00 | 3.10 | 2.19 |
| **338** | **1.36** | **47.43** | **3.23** | **4.47** | **2.12** | **<LOQ** | **<LOQ** | **0.51** | **8.37** | **5.90** |
| **339** | **0.30** | **41.81** | **291.48** | **258.31** | **78.16** | **<LOQ** | **<LOQ** | **6.81** | **12.76** | **8.18** |
| 340 | 3.48 | 29.61 | 33.75 | 54.06 | 29.48 | 27.17 | 63.07 | 2.26 | 2.64 | 2.01 |
| 344 | 0.28 | 11.18 | 8.84 | 11.88 | 6.74 | 226.07 | <LOQ | 1.87 | 2.42 | 1.90 |
| P3-1 | 4.18 | 31.61 | <LOQ | <LOQ | <LOQ | 23.86 | 90.89 | 0.88 | 11.21 | 8.73 |
| P3-2 | 1.87 | 3.34 | 10.43 | 24.63 | 14.57 | 40.67 | <LOQ | 1.25 | 1.35 | 0.80 |
| P3-3 | 5.50 | 37.16 | 43.62 | 72.59 | 46.84 | 12.38 | 36.16 | 0.32 | 3.90 | 2.72 |
| P3-4 | 1.39 | 20.90 | 112.76 | 169.04 | 88.63 | 141.01 | <LOQ | 3.56 | 5.37 | 3.77 |
| P3-5 | 3.86 | 69.91 | 54.53 | 49.90 | 11.09 | 17.62 | <LOQ | 0.63 | 6.31 | 4.11 |
| P3-6 | 6.55 | 21.23 | 59.83 | 51.93 | 11.93 | 54.36 | 109.59 | 1.75 | 1.38 | 1.00 |
| P3-7 | 1.42 | 24.94 | 64.91 | 76.26 | 32.79 | 65.51 | <LOQ | 1.65 | 4.37 | 2.85 |
| P3-8 | 3.47 | 63.13 | <LOQ | <LOQ | <LOQ | 24.06 | 67.67 | <LOQ | 6.46 | 4.41 |

**Table S3**

| **Weight [mg] (n=3)** | **SD** | **CV [%]** | **Total Protein Concentration [µg/µl] (n=3)** | **SD** | **CV [%]** |
| --- | --- | --- | --- | --- | --- |
| 25.3 | 5.69 | 22.6 | 0.97 | 0.21 | 21.9 |
| 50.1 | 3.32 | 6.6 | 3.22 | 0.21 | 6.4 |
| 74.5 | 5.53 | 7.4 | 5.25 | 0.55 | 10.5 |
| 100.4 | 7.68 | 7.7 | 6.60 | 0.32 | 4.9 |
| 125.1 | 6.22 | 5.0 | 9.14 | 0.29 | 3.2 |
| 150.1 | 3.56 | 2.4 | 10.34 | 0.54 | 5.2 |

**Table S4**

| **Protein Number** | **Peptide Sequence** | **Precursor Ion** | **Product Ion** | Qualified/**Quantified** ion |
| --- | --- | --- | --- | --- |
| P01009 | AVLTIDEK.light | 444.8 | 718.4 | **y6** |
| P01009 | AVLTIDEK.light | 444.8 | 605.3 | y5 |
| P01009 | AVLTIDEK.light | 444.8 | 504.3 | y4 |
| P01009 | AVLTIDEK.heavy | 448.8 | 726.4 | **y6** |
| P01009 | AVLTIDEK.heavy | 448.8 | 613.3 | y5 |
| P01010 | AVLTIDEK.heavy | 448.8 | 512.3 | y4 |
| P01876 | TPLTATLSK.heavy | 470.3 | 741.5 | y7 |
| P01876 | TPLTATLSK.heavy | 470.3 | 527.3 | y5 |
| P01876 | TPLTATLSK.heavy | 470.3 | 419.8 | **y8** |
| P01876 | TPLTATLSK.heavy | 470.3 | 312.2 | b3 |
| P01876 | TPLTATLSK.heavy | 470.3 | 242.2 | y2 |
| P01876 | TPLTATLSK.light | 466.3 | 733.4 | y7 |
| P01876 | TPLTATLSK.light | 466.3 | 519.3 | y5 |
| P01876 | TPLTATLSK.light | 466.3 | 415.8 | **y8** |
| P01876 | TPLTATLSK.light | 466.3 | 312.2 | b3 |
| P01876 | TPLTATLSK.light | 466.3 | 234.1 | y2 |
| P01877 | DASGATFTWTPSSGK.light | 756.9 | 1111.5 | y10 |
| P01877 | DASGATFTWTPSSGK.light | 756.9 | 1010.5 | y9 |
| P01877 | DASGATFTWTPSSGK.light | 756.9 | 863.4 | y8 |
| P01877 | DASGATFTWTPSSGK.light | 756.9 | 762.4 | y7 |
| P01877 | DASGATFTWTPSSGK.light | 756.9 | 475.3 | **y5** |
| P01877 | DASGATFTWTPSSGK.heavy | 760.9 | 1119.6 | y10 |
| P01877 | DASGATFTWTPSSGK.heavy | 760.9 | 1018.5 | y9 |
| P01877 | DASGATFTWTPSSGK.heavy | 760.9 | 871.4 | y8 |
| P01877 | DASGATFTWTPSSGK.heavy | 760.9 | 770.4 | y7 |
| P01877 | DASGATFTWTPSSGK.heavy | 760.9 | 483.3 | **y5** |
| P01877 | SAVQGPPER.light | 470.7 | 782.4 | y7 |
| P01877 | SAVQGPPER.light | 470.7 | 683.3 | y6 |
| P01877 | SAVQGPPER.light | 470.7 | 555.3 | **y5** |
| P01877 | SAVQGPPER.light | 470.7 | 498.3 | y4 |
| P01877 | SAVQGPPER.light | 470.7 | 401.2 | y3 |
| P01877 | SAVQGPPER.light | 470.7 | 258.1 | b3 |
| P01877 | SAVQGPPER.heavy | 475.8 | 792.4 | y7 |
| P01877 | SAVQGPPER.heavy | 475.8 | 693.4 | y6 |
| P01877 | SAVQGPPER.heavy | 475.8 | 565.3 | **y5** |
| P01877 | SAVQGPPER.heavy | 475.8 | 508.3 | y4 |
| P01877 | SAVQGPPER.heavy | 475.8 | 411.2 | y3 |
| P01877 | SAVQGPPER.heavy | 475.8 | 258.1 | b3 |
| P05109 | ALNSIIDVYHK.light | 636.9 | 887.5 | y7 |
| P05109 | ALNSIIDVYHK.light | 636.9 | 774.4 | **y6** |
| P05109 | ALNSIIDVYHK.light | 636.9 | 661.3 | y5 |
| P05109 | ALNSIIDVYHK.light | 636.9 | 284.2 | y2 |
| P05109 | ALNSIIDVYHK.light | 636.9 | 299.2 | b3 |
| P05109 | ALNSIIDVYHK.heavy | 640.9 | 895.5 | y7 |
| P05109 | ALNSIIDVYHK.heavy | 640.9 | 782.4 | **y6** |
| P05109 | ALNSIIDVYHK.heavy | 640.9 | 669.3 | y5 |
| P05109 | ALNSIIDVYHK.heavy | 640.9 | 292.2 | y2 |
| P05109 | ALNSIIDVYHK.heavy | 640.9 | 299.2 | b3 |
| P05164 | VVLEGGIDPILR.light | 640.9 | 840.5 | **y8** |
| P05164 | VVLEGGIDPILR.light | 640.9 | 498.3 | y4 |
| P05164 | VVLEGGIDPILR.light | 640.9 | 312.2 | b3 |
| P05164 | VVLEGGIDPILR.heavy | 645.9 | 850.5 | **y8** |
| P05164 | VVLEGGIDPILR.heavy | 645.9 | 508.3 | y4 |
| P05164 | VVLEGGIDPILR.heavy | 645.9 | 312.2 | b3 |
| P05164 | VVLEGGIDPILR.light | 427.6 | 498.3 | y4 |
| P05164 | VVLEGGIDPILR.light | 427.6 | 249.7 | y4 |
| P05164 | VVLEGGIDPILR.heavy | 430.9 | 508.3 | y4 |
| P05164 | VVLEGGIDPILR.heavy | 430.9 | 254.7 | y4 |
| P06702 | DLQNFLK.light | 439.2 | 649.4 | **y5** |
| P06702 | DLQNFLK.light | 439.2 | 521.3 | y4 |
| P06702 | DLQNFLK.light | 439.2 | 407.3 | y3 |
| P06702 | DLQNFLK.light | 439.2 | 260.2 | y2 |
| P06702 | DLQNFLK.light | 439.2 | 229.1 | b2 |
| P06702 | DLQNFLK.heavy | 443.2 | 657.4 | **y5** |
| P06702 | DLQNFLK.heavy | 443.2 | 529.3 | y4 |
| P06702 | DLQNFLK.heavy | 443.2 | 415.3 | y3 |
| P06702 | DLQNFLK.heavy | 443.2 | 268.2 | y2 |
| P06702 | DLQNFLK.heavy | 443.2 | 229.1 | b2 |
| P06702 | LGHPDTLNQGEFK.light | 485.9 | 722.3 | **y6** |
| P06702 | LGHPDTLNQGEFK.light | 485.9 | 608.3 | y5 |
| P06702 | LGHPDTLNQGEFK.light | 485.9 | 480.2 | y4 |
| P06702 | LGHPDTLNQGEFK.light | 485.9 | 294.2 | y2 |
| P06702 | LGHPDTLNQGEFK.light | 485.9 | 621.3 | b6 |
| P06702 | LGHPDTLNQGEFK.heavy | 488.6 | 730.4 | **y6** |
| P06702 | LGHPDTLNQGEFK.heavy | 488.6 | 616.3 | y5 |
| P06702 | LGHPDTLNQGEFK.heavy | 488.6 | 488.3 | y4 |
| P06702 | LGHPDTLNQGEFK.heavy | 488.6 | 302.2 | y2 |
| P06702 | LGHPDTLNQGEFK.heavy | 488.6 | 621.3 | b6 |
| P10153 | DPPQYPVVPVHLDR.light | 544.6 | 736.4 | y6 |
| P10153 | DPPQYPVVPVHLDR.light | 544.6 | 710.4 | y12 |
| P10153 | DPPQYPVVPVHLDR.light | 544.6 | 368.7 | y6 |
| P10153 | DPPQYPVVPVHLDR.light | 544.6 | 473.9 | **y12** |
| P10153 | DPPQYPVVPVHLDR.light | 544.6 | 213.8 | y5 |
| P10153 | DPPQYPVVPVHLDR.heavy | 548.0 | 746.4 | y6 |
| P10153 | DPPQYPVVPVHLDR.heavy | 548.0 | 715.4 | y12 |
| P10153 | DPPQYPVVPVHLDR.heavy | 548.0 | 373.7 | y6 |
| P10153 | DPPQYPVVPVHLDR.heavy | 548.0 | 477.3 | **y12** |
| P10153 | DPPQYPVVPVHLDR.heavy | 548.0 | 217.1 | y5 |
| P12724 | NQNTFLR.light | 446.7 | 778.4 | y6 |
| P12724 | NQNTFLR.light | 446.7 | 650.4 | **y5** |
| P12724 | NQNTFLR.light | 446.7 | 536.3 | y4 |
| P12724 | NQNTFLR.light | 446.7 | 435.3 | y3 |
| P12724 | NQNTFLR.light | 446.7 | 288.2 | y2 |
| P12724 | NQNTFLR.heavy | 451.7 | 788.4 | y6 |
| P12724 | NQNTFLR.heavy | 451.7 | 660.4 | **y5** |
| P12724 | NQNTFLR.heavy | 451.7 | 546.3 | y4 |
| P12724 | NQNTFLR.heavy | 451.7 | 445.3 | y3 |
| P12724 | NQNTFLR.heavy | 451.7 | 298.2 | y2 |

**Table S5**

| **Peptide Sequence** | **Peptide concentration in sample[nM] (n=3)** | | | | | | | | | | | |
| --- | --- | --- | --- | --- | --- | --- | --- | --- | --- | --- | --- | --- |
|  | **1h** | **CV [%]** | **3h** | **CV [%]** | **5h** | **CV [%]** | **17h** | **CV [%]** | **20h** | **CV [%]** | **24h** | **CV [%]** |
| AVLTIDEK | 1270.7 | 14.7 | 1096.9 | 2.3 | 1406.4 | 5.2 | 1119.9 | 8.0 | 1161.5 | 24.1 | 1140.4 | 2.1 |
| TPLTATLSK | 341.2 | 10.4 | 366.2 | 6.7 | 405.7 | 6.4 | 334.1 | 2.0 | 320.9 | 5.3 | 352.5 | 4.6 |
| SAVQGPPER | 1010.9 | 5.7 | 1073.5 | 8.1 | 1086.2 | 5.8 | 1032.2 | 1.7 | 1046.6 | 9.0 | 1056.0 | 4.4 |
| DASGATFTWTPSSGK | 868.3 | 2.5 | 927.8 | 17.7 | 974.3 | 16.7 | 963.2 | 20.7 | 902.6 | 6.8 | 964.6 | 13.4 |
| NQNTFLR | 4.9 | 8.0 | 6.8 | 8.0 | 6.7 | 6.3 | 7.0 | 5.5 | 6.6 | 7.4 | 7.7 | 4.6 |
| DPPQYPVVPVHLDR | 10.2 | 16.3 | 12.1 | 15.0 | 11.7 | 14.4 | 14.6 | 10.3 | 15.0 | 10.0 | 11.6 | 23.9 |
| VVLEGGIDPILR | 33.6 | 58.7 | 53.3 | 14.1 | 77.2 | 13.0 | 34.7 | 63.1 | 19.5 | 38.0 | 32.1 | 3.1 |
| ALNSIIDVYHK | 955.9 | 13.0 | 920.0 | 3.8 | 1022.2 | 7.2 | 1098.7 | 8.1 | 981.5 | 20.2 | 1170.4 | 1.6 |
| DLQNFLK | 1531.2 | 24.5 | 1496.3 | 9.7 | 1764.3 | 9.2 | 1459.5 | 28.0 | 1407.2 | 21.5 | 1732.2 | 9.5 |
| LGHPDTLNQGEFK | 902.3 | 7.3 | 944.2 | 5.1 | 1030.0 | 9.6 | 1049.4 | 3.1 | 1129.0 | 11.5 | 1220.3 | 11.5 |

**Table S6**

| **Protein** | **Peptide Sequence** | **Protein Concentration µg/mg of total protein (n=6)** | | | | | | **Intraday precision** | |
| --- | --- | --- | --- | --- | --- | --- | --- | --- | --- |
|  |  | **1** | **2** | **3** | **4** | **5** | **6** | **Average [µg/mg]** | **CV [%]** |
| A1AT-1 | AVLTIDEK | 17.8 | 18.6 | 17.7 | 17.8 | 15.7 | 14.1 | 17.0 | 10.0 |
| IGHA1 | TPLTATLSK | 3.5 | 3.0 | 3.5 | 3.0 | 2.9 | 2.6 | 3.1 | 11.4 |
| IGHA1+2 | SAVQGPPER | 9.4 | 8.5 | 8.3 | 8.1 | 7.8 | 7.6 | 8.3 | 7.6 |
| IGHA2 | DASGATFTWTPSSGK | 5.4 | 5.7 | 6.6 | 6.5 | 5.9 | 6.6 | 6.1 | 8.3 |
| ECP | NQNTFLR | 0.02 | 0.02 | 0.02 | 0.02 | 0.02 | 0.02 | 0.02 | 7.3 |
| EDN | DPPQYPVVPVHLDR | 0.04 | 0.03 | 0.05 | 0.03 | 0.04 | 0.03 | 0.04 | 17.8 |
| MPO | VVLEGGIDPILR | 0.08 | 0.09 | 0.08 | 0.08 | 0.06 | 0.09 | <LOQ (0.08) | 12.1 |
| CAL1 | ALNSIIDVYHK | 3.3 | 2.8 | 2.9 | 2.6 | 2.3 | 2.6 | 2.8 | 11.9 |
| CAL2 | DLQNFLK | 2.6 | 2.7 | 2.7 | 2.2 | 2.5 | 2.2 | 2.5 | 9.6 |
|  | LGHPDTLNQGEFK | 2.9 | 3.0 | 2.7 | 2.7 | 2.4 | 2.6 | 2.7 | 7.6 |

**Table S7**

| **Protein** | **Peptide Sequence** | **1st day** | | **2nd day** | | **3rd day** | | **Interday precision** | |
| --- | --- | --- | --- | --- | --- | --- | --- | --- | --- |
|  |  | **Average [µg/mg]** | **CV [%]** | **Average [µg/mg]** | **CV [%]** | **Average [µg/mg]** | **CV [%]** | **Average [µg/mg]** | **CV [%]** |
| A1AT-1 | AVLTIDEK | 17.0 | 10.0 | 19.4 | 3.8 | 21.9 | 16.0 | 19.4 | 12.8 |
| IGHA1 | TPLTATLSK | 3.1 | 11.4 | 3.8 | 16.2 | 5.4 | 20.1 | 4.1 | 28.9 |
| IGHA1+2 | SAVQGPPER | 8.3 | 7.6 | 8.8 | 8.3 | 11.1 | 13.9 | 9.4 | 16.2 |
| IGHA2 | DASGATFTWTPSSGK | 6.1 | 8.3 | 6.4 | 13.3 | 6.6 | 9.8 | 6.4 | 3.6 |
| ECP | NQNTFLR | 0.02 | 7.3 | 0.02 | 8.9 | 0.02 | 16.2 | 0.02 | 8.5 |
| EDN | DPPQYPVVPVHLDR | 0.04 | 17.8 | 0.04 | 13.1 | 0.04 | 18.8 | 0.04 | 9.6 |
| MPO | VVLEGGIDPILR | <LOQ (0.08) | 12.1 | <LOQ (0.06) | 13.1 | <LOQ (0.04) | 17.7 | <LOQ (0.06) | 29.9 |
| CAL1 | ALNSIIDVYHK | 2.8 | 11.9 | 2.0 | 9.1 | 2.4 | 12.2 | 2.4 | 15.8 |
| CAL2 | DLQNFLK | 2.5 | 9.6 | 1.8 | 9.4 | 2.3 | 23.8 | 2.2 | 15.5 |
|  | LGHPDTLNQGEFK | 2.7 | 7.6 | 2.3 | 7.8 | 2.4 | 12.6 | 2.5 | 9.3 |

**Table S8**

| **Protein** | **Peptide Sequence** | **Protein Concentration**  **[µg/mg of total protein]** | **CV [%]** |
| --- | --- | --- | --- |
|  |  |  |  |
| IGHA1 | TPLTATLSK | 5.91 | 15.8 |
| IGHA1+IGHA2 | SAVQGPPER | 10.36 | 13.8 |
| IGHA2 | DASGATFTWTPSSGK | 7.78 | 17.2 |
| A1AT-1 | AVLTIDEK | 53.98 | 16.6 |
| CAL1 | ALNSIIDVYHK | 5.19 | 18.1 |
| CAL2 | LGHPDTLNQGEFK | 3.21 | 20.6 |
|  | DLQNFLK | 5.30 | 13.2 |
| MPO | VVLEGGIDPILR | 0.53 | 10.0 |
| ECP | NQNTFLR | 0.02 | 10.1 |
| EDN | DPPQYPVVPVHLDR | 0.04 | 25.1 |
